# Resistance to anti-HER2 therapy associated with the *TSC2* nonsynonymous variant c.4349 C > G (p.Pro1450Arg) is reversed by CDK4/6 inhibitor in HER2-positive breast cancer

**DOI:** 10.1038/s41523-023-00542-1

**Published:** 2023-05-09

**Authors:** Ziyan Yang, Jianguo Feng, Ji Jing, Yuan Huang, Wei-Wu Ye, Lei Lei, Xiao-Jia Wang, Wen-Ming Cao

**Affiliations:** 1 Department of Breast Medical Oncology, Cancer Hospital of the University of Chinese Academy of Sciences (Zhejiang Cancer Hospital), Hangzhou, 310022 China; 2 Cancer Center, Department of Medical Oncology, Zhejiang Provincial People’s Hospital, Affiliated People’s Hospital, Hangzhou Medical College, Hangzhou, Zhejiang 310014 China; 3 Institute of Cancer and Basic Medicine (ICBM), Chinese Academy of Sciences, Hangzhou, 310063 China; 4 Zhejiang Cancer Institute, Cancer Hospital of the University of Chinese Academy of Sciences (Zhejiang Cancer Hospital), Hangzhou, 310022 China

**Keywords:** Breast cancer, Cancer therapeutic resistance

## Abstract

HER2-positive breast cancer patients carrying the germline *TSC2* nonsynonymous variant c.4349 C > G (p.Pro1450Arg) are resistant to anti-HER2 therapy. Multi-predictor in silico analysis reveals that this variant is deleterious. We explore the potential mechanism of this *TSC2* variant and investigate methods for overcoming anti-HER2 resistance. *TSC2* c.4349 C > G reverses the inhibitory effect on mTOR and downstream signaling by increasing TSC2 phosphorylation at Thr1462 and confers significant lapatinib resistance in vitro and in vivo. The combination of lapatinib and the CDK4/6 inhibitor palbociclib inhibits cyclin D1/CDK4/Rb alternative pathway and TSC2 phosphorylation, thereby partially attenuating mTOR activity and inducing *TSC2*-mutant cell blockage at G1/G0. In in vitro and xenograft models, palbociclib+lapatinib shows higher anti-tumor activity than monotherapy and overcomes the resistance of the *TSC2* c.4349 C > G-related variant to anti-HER2 therapy. We reveal a new mechanism of resistance to anti-HER2 therapy and provide a strategy to increase the efficiency of anti-HER2 therapy in HER2-positive breast cancer.

## Introduction

Breast cancer is one of the most common forms of cancer, with the highest incidence rates, among women^1,2^. Estrogen receptor (ER), progesterone receptor (PR), and human epidermal growth factor receptor 2 (HER2) are important biological markers of breast cancer^3^. Currently, several HER2-targeted therapies are used to treat HER2-positive breast cancer. Trastuzumab is a humanized anti-HER2 antibody targeting the extracellular domain of HER2^4^. Lapatinib is an oral reversible tyrosine kinase inhibitor that specifically inhibits HER1 and HER2 and the activity of their downstream signaling pathways^5^. Mutations in the genes involved in the HER2 pathway are the driving forces underlying resistance to anti-HER2 therapy^6^. The HER2/AKT/mammalian target of rapamycin (mTOR) signal node is one of the most extensive signaling pathways involved in human cancers^7^ and participates in the control of cell metabolism, movement, proliferation, growth, survival, and other cellular processes^8^.

Germline pathogenic mutations in tuberous sclerosis 1 (*TSC1*) or *TSC2* causes tuberous sclerosis, an autosomal dominant genetic disease characterized by the development of hamartomas in multiple organs or tissues, including the skin, brain, eyes, lungs, heart, and kidneys^9^. TSC1 and TSC2 exist in the cells as the TSC1/TSC2 complex, which acts as a GTPase-activating protein for the GTPase homolog enriched in the brain (Rheb)^10^. TSC2 can convert the GTP form of Rheb into the GDP form, thereby inhibiting the activity of Rheb and blocking the mTOR pathway. Functional loss of TSC2 results in constitutive activation of Rheb and its downstream mTOR signaling^11^. Palbociclib, a cyclin-dependent kinase (CDK) inhibitor, can inhibit the cyclin D1/CDK4/Retinoblastoma (Rb) alternative pathway and indirectly reduce the phosphorylation level of Thr1462 in TSC2, thereby suppressing the activity of mTOR and alleviating feedback inhibition of epidermal growth factor receptor (EGFR) family kinases^12^. This process improves the sensitivity of the cells to the effects of anti-HER2 therapy. Thus, targeted therapy (trastuzumab or lapatinib) combined with CDK4/6 inhibitors is a promising strategy for the treatment of HER2-positive breast cancer.

In our clinical practice, a HER2-positive patient with the *TSC2* (NM_000548) c.4349 C > G (p.Pro1450Arg) germline nonsynonymous variant was found to be resistant to trastuzumab (administered as part of a neoadjuvant therapy). Thus, in this study, we aimed to construct stable *TSC2* mutant and wild-type cell lines to investigate the mechanism of action of this anti-HER2-resistant variant. We also verified our results using cell-derived xenografts and clinical specimens and identified a novel strategy to improve the efficacy of anti-HER2 therapy in HER2-positive breast cancer.

## Results

### Resistance to anti-HER2 therapy in a breast cancer patient with *TSC2* c.4349 C > G (p. Pro1450Arg) germline variant

**Fig. 1:**
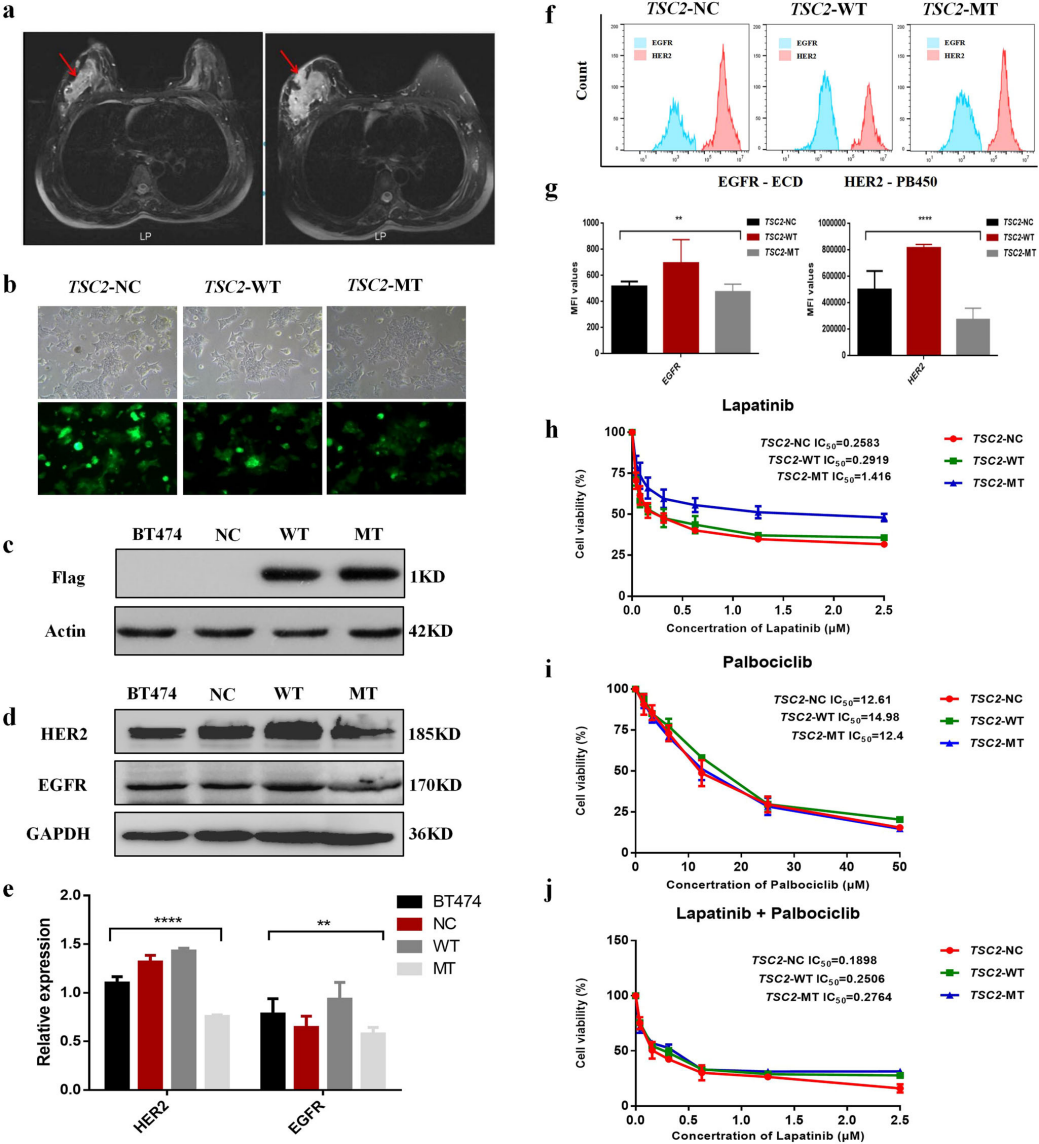
Magnetic resonance imaging (MRI) reveals a clinical response to neoadjuvant therapy containing trastuzumab in a breast cancer patient with the *TSC2* c.4349 C > G (p.Pro1450Arg) variant and mutant TSC2 conferring resistance to lapatinib. **a** Left: Breast MRI scan captured before trastuzumab treatment initiation. Right: Follow-up image captured after trastuzumab treatment initiation, showing considerable tumor enlargement after four treatment cycles. **b** Three groups of lentiviruses were added to the BT474 culture to validate the transfection efficacy using fluorescence microscopy. Images in **b** are at ×20 magnification. Scale bar for each image is 1 cm. **c** Expression of the FLAG tag in the *TSC2*-NC, *TSC2*-WT, and *TSC2*-MT groups was determined via western blotting. **d** Expression of EGFR and HER2 in BT474, *TSC2*-NC, *TSC2*-WT, and *TSC2*-MT cells was determined via western blotting. **e** Relative expression of EGFR and HER2 in BT474, *TSC2*-NC, *TSC2*-WT, and *TSC2*-MT. **(f)** Expression of EGFR and HER2 in *TSC2*-NC, *TSC2*-WT, and *TSC2*-MT cells was determined by flow cytometry. **g** Mean fluorescence intensity (MFI) values of EGFR and HER2 in *TSC2*-NC, *TSC2*-WT, and *TSC2*-MT cells. **h** IC_50_ values of lapatinib for the *TSC2*-NC, *TSC2*-WT, and *TSC2*-MT groups are 0.2503 μM, 0.2919 μM, and 1.416 μM, respectively. Overexpression of *TSC2*-MT enhanced lapatinib resistance compared with that of *TSC2*-WT (*P* < 0.001). **i** Inhibition curve of the CDK4/6 inhibitor palbociclib. **j** Under combined treatment with palbociclib and lapatinib, palbociclib concentration was fixed (IC_20_ = 5 μM). IC_50_ values of lapatinib for the *TSC2*-NC, *TSC2-*WT, and *TSC2-*MT groups are 0.1838 μM, 0.2506 μM, and 0.2764 μM, respectively. Data are shown as mean± (Standard Deviation) SD; Data in (E, G, H, I, J) were analyzed with one-way ANOVA. Error bars are standard error of mean (SEM). ^**^P < 0.05, ^****^P < 0.0001.

The patient, diagnosed with Luminal B (HER2 positive) breast cancer, showed progress after anti-HER2 neoadjuvant treatment (including trastuzumab) according to the Response Evaluation Criteria in Solid Tumors version 1.1 guidelines (Fig. 1a). *TSC2* germline nonsynonymous variant (NM_000548.5, rs45517338) c.4349 C > G (p.Pro1450Arg) was identified in the patient by a 98-gene panel sequencing assay (genes are listed in Supplementary Table 1), which was demonstrated conflicting interpretations of pathogenicity (either “likely benign” or “of uncertain significance”). The highest-population minor allele frequency of guanine (G) was lower than 0.01, as determined by 1000 Genomes Phase 3, ESP, and gnomAD. The variant was predicted as being damaging and probably damaging by SFIT and PolyPhen-2 in silico analysis, respectively. Whole-exome sequencing suggested that none of the other somatic alterations were associated with resistance to anti-HER2 treatment (Supplementary Table 2).

### Mutant *TSC2* confers resistance to lapatinib

As the patient carrying the *TSC2* c.4349 C > G variant was resistant to trastuzumab, we constructed plasmids to investigate the role of the variant in mediating resistance to anti-HER2 therapy using the BT474 cell line. We first observed transfection efficiency using a fluorescence microscope (Fig. 1b). The expression of FLAG-tagged target protein was verified using western blotting (Fig. 1c). EGFR and HER2 expression were detected by western blotting in four groups of cells: BT474, *TSC2*-NC, *TSC2*-WT, and *TSC2*-MT (Fig. 1d). As shown in Fig. 1e, the expression of HER2 was significantly higher than that of EGFR in all four groups of cells, and the expression levels of HER2 and EGFR were significantly lower in the MT group than in the other three groups (*n* = 4, F = 107.7, *P* = 0.0001 and F = 4.21, *P* = 0.046, respectively; one-way ANOVA). To differentiate the expression of EGFR and HER2 in the NC, WT, and MT groups, we quantified the expression of these growth factor receptors in the different cell lines, using flow cytometry (Fig. 1f). The mean fluorescence intensity (MFI) values of EGFR in the NC, WT, and MT groups were 516.7 ± 15.3, 695 ± 72.5, and 476 ± 22.5, respectively. There were significant differences in the MFIs among these three groups (*n* = 3, F = 20.28, *P* = 0.0021; one-way ANOVA). The MFI values of HER2 were 5.02 × 10^5^ ± 55770, 8.32 × 10^5^ ± 15620, and 2.76 × 10^5^ ± 34100, respectively (Fig. 1g). The MFI was significantly different among the three groups (*n* = 3, F = 151.8, *P* = 0.0001; one-way ANOVA). The inhibitory effect of the drugs on cell growth was dependent on the drug concentration. The half-maximal inhibitory concentration (IC_50_) values of lapatinib in the NC, WT, and MT groups were 0.2583, 0.2919, and 1.416 μM, respectively. Compared with the WT and NC groups, the MT group showed a significantly higher drug resistance (*n* = 3, F = 36.25, *P* = 0.0001; one-way ANOVA; Fig. 1h). However, no significant difference was noted with respect to palbociclib monotherapy in these three groups of cells (*n* = 3, *P* > 0.05; one-way ANOVA; Fig. 1i). When lapatinib was combined with palbociclib, the concentration of palbociclib was fixed (IC_20_ = 5 μM). The IC_50_ values of lapatinib in the WT and MT groups were 0.2506 and 0.2764 μM, respectively. Furthermore, there was no significant difference among the three groups of cells in the combination group (*n* = 3, *P* > 0.05; one-way ANOVA; Fig. 1j).

Finally, we compared the effects of the two drugs as a function of the concentrations of lapatinib, palbociclib, and lapatinib+palbociclib. The drug combination index (CI) was calculated for the WT and MT groups when the two drugs were combined. The CI of lapatinib+palbociclib in both groups was <1, indicating a synergistic effect. Lapatinib demonstrated a synergistic sensitization effect at concentrations lower than 0.25 μmol/L. The CI values at IC_50_ for the WT and MT groups under the two-drug combination condition (for lapatinib alone, WT was 0.2608 μM and MT was 0.2764 μM) were 0.502 and 0.312, respectively. The synergistic sensitization effect of the two drugs was more evident in the MT group than in the WT group.

### Palbociclib induces G1 arrest in HER2-amplified BT474 cells

**Fig. 2:**
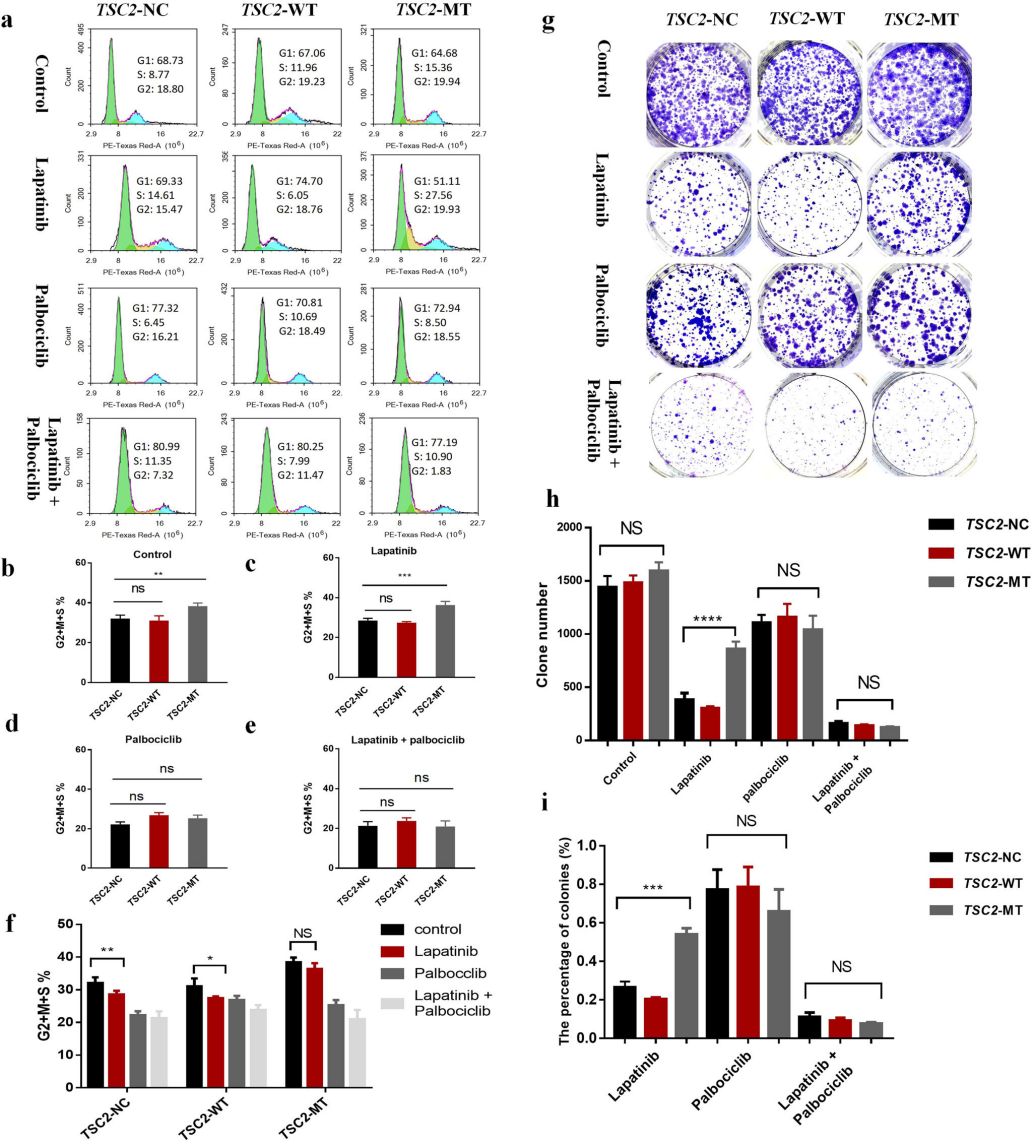
Cell cycle and colony-forming ability of BT474 cell lines treated with lapatinib and/or palbociclib. **a** Cell cycle analysis of BT474 cell lines treated with lapatinib and/or palbociclib. **b** Proportion of G2 + M + S cells in the *TSC2*-MT group increased compared with that in the *TSC2*-WT and *TSC2*-NC groups without treatment (*n* = 3, F = 12.58, *P* = 0.0071; one-way ANOVA). **c** *TSC2*-MT group showed an increased proportion of cells in the G2 + M + S stages compared to the other two groups on lapatinib treatment (*n* = 3, F = 42.19, *P* = 0.0003; one-way ANOVA). **d** Three groups of cells showed a similar proportion of cells in the G2 + M + S stages upon palbociclib treatment. **e** Under the lapatinib + palbociclib treatment, the proportion of cells in the G2 + M + S stages was not significantly different among the three groups. **f** Cell cycle comparison under control, lapatinib, palbociclib, or lapatinib+palbociclib treatments within the same cell line. **g** Representative images of colony staining for the three BT474 cell lines treated with control, lapatinib, palbociclib, and lapatinib+palbociclib. **h** Colony count comparison in the three groups treated with lapatinib and/or palbociclib. The *TSC2*-MT group showed significantly higher colony counts than the *TSC2*-WT and *TSC2*-NC groups on lapatinib treatment (*n* = 3, F = 85.54, *P* = 0.0001; one-way ANOVA). However, under conditions involving stress induced by both lapatinib and palbociclib, the colony-forming ability of the three groups did not significantly differ (*n* = 3, *F* = 3.5, *P* = 0.098; one-way ANOVA). **i** Clone number rate comparisons relative to the control after lapatinib and/or palbociclib treatment. In the lapatinib group, the clone number rate in the *TSC2*-MT group was higher than that in the other two groups (*n* = 3, *F* = 92.83, *P* = 0.0004; one-way ANOVA). Data are shown as mean ± SD; data in **b**, **c**, **d**, **e**, **f** and **I** were analyzed with two-tailed *T* test and one-way ANOVA. Error bars are SEM. ^*^P < 0.05, ^**^P < 0.005, ^***^P < 0.001, ^****^P < 0.0001.

The results of the cell cycle assay are shown in Fig. 2a. In the untreated group, the proportion of cells in the G2 + S + M phase of the *TSC2*-MT group (38.33 ± 1.53) was significantly higher than that of the other two groups (NC: 32.10 ± 1.71, WT: 31.07 ± 2.41; *n* = 3, F = 12.58, *P* = 0.0071; one-way ANOVA; Fig. 2b). When exposed to lapatinib, the MT group (36.34 ± 1.82) showed a significantly higher proportion of cells in the G2 + M + S stages than that in the WT and NC groups (WT: 27.43 ± 0.57, NC: 28.51 ± 0.57; *n* = 3, F = 42.19, *P* = 0.0003; one-way ANOVA). Collectively, *TSC2*-MT was more resistant to lapatinib than *TSC2*-WT, demonstrated by an increase in cell proliferation (Fig. 2c). The proportion of G2 + M + S cells in the MT group was significantly lower after palbociclib monotherapy (25.27 ± 1.60) than after lapatinib monotherapy (36.34 ± 1.82, *n* = 2, *t* = 7.93, *P* = 0.0014; *T* test). After incubation with palbociclib, no significant difference was observed among the three groups (Fig. 2d). Moreover, when compared to the results observed after the use of lapatinib, the proportion of cells in the G2 + M + S proliferation phase was significantly reduced upon combined treatment with lapatinib and palbociclib (Fig. 2e). Compared with that in the control group, lapatinib treatment could reduce the proportion of G2 + M + S phase cells in the NC and WT groups (*n* = 2, *t* = 2.9, *P* = 0.004; *t* = 2.78, *P* = 0.049; T test; Fig. 2f), although the inhibition of the cell cycle by lapatinib was not significant in the MT group. However, there was no difference in the proportions of G2 + S + M phase cells in all three groups after treatment with palbociclib (Fig. 2d). These results indicated that the combination of the two drugs could induce blockage of most cells at G1/G0, thereby slowing down cell proliferation.

### Combination of lapatinib and palbociclib effectively reduces the ability of cells to form colonies in vitro

We conducted colony formation assays to determine the antitumorigenic effect of lapatinib, palbociclib, and a combination of the two drugs on the three groups of cells (Fig. 2g). There was no significant difference in the number of clones among the three control groups. We then explored the colony-forming ability of the cells under conditions involving lapatinib-induced stress. No significant difference was observed in the number of clones between the *TSC2*-WT (301.7 ± 20.79) and *TSC2*-NC (382.3 ± 63.2) groups (*t* = 2.1, *P* = 0.078; *T* test). However, the number of colonies was higher in the *TSC2-*MT (625.3 ± 31.5) group than in the other two groups (*n* = 3, F = 85.54, *P* = 0.0001; one-way ANOVA), indicating that the MT group showed significant resistance to lapatinib compared to the WT and NC groups. In the case of palbociclib treatment, no significant difference was observed among the three cell groups. In the case of combination treatment, no significant difference was observed among the NC, WT, and MT groups (129 ± 5.77, 152 ± 18.1, and 137.3 ± 35.9.4, respectively; *n* = 3, F = 3.5, *P* = 0.098; one-way ANOVA; Fig. 2h). Owing to the difference in the transfection efficiency of the three groups of cells, the number of clones treated with lapatinib, palbociclib, and the two-drug combination was compared with that of the control group. The percentage of colonies relative to the control in lapatinib-treated cells for the MT group was significantly higher than that for the other two groups (*n* = 3, F = 92.83, *P* = 0.0004; one-way ANOVA). However, there was no significant difference in the percentage of cells after palbociclib treatment (*n* = 3, F = 1.24, *P* = 0.3347; one-way ANOVA; Fig. 2i). These results indicate that palbociclib increased sensitivity to lapatinib.

### The HER2 pathway is associated with the phosphorylation levels of TSC2

**Fig. 3:**
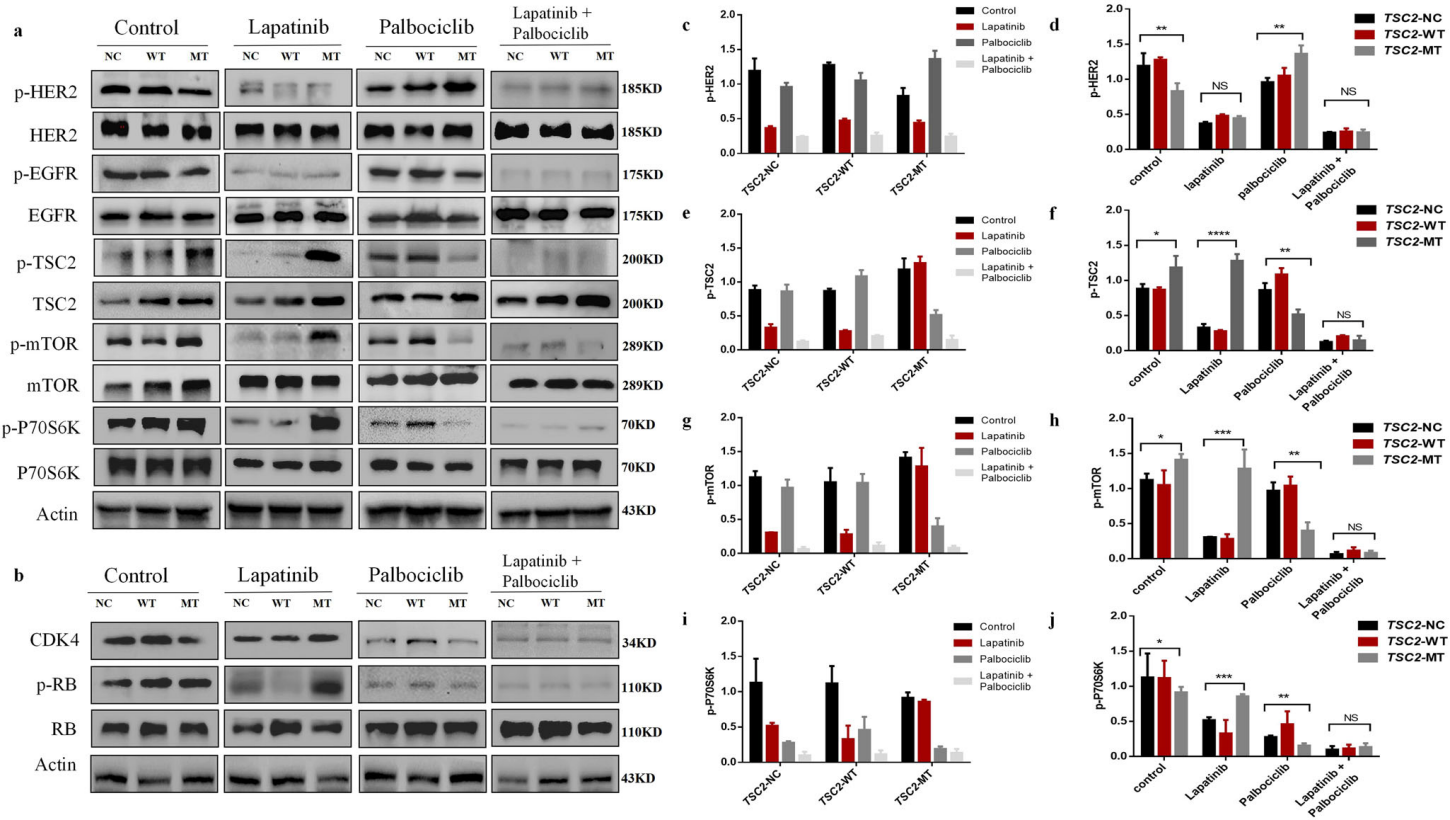
Expression of proteins involved in the HER2/AKT/mTOR and cyclinD1/CDK4 signaling pathways in BT474 cell lines treated with lapatinib and/or palbociclib. **a** Western blot analysis of proteins involved in the HER2/AKT/mTOR signaling pathway after treatment with lapatinib and/or palbociclib for 48 h. **b** Western blot analysis of proteins involved in the cyclinD1/CDK4 signaling pathway after treatment with lapatinib and/or palbociclib for 48 h. **c** p-HER2 expression levels under treatment with different drugs in the same cell. **d** p-HER2 expression levels in *TSC2*-NC, WT, and MT groups treated with the same drugs. **e** p-TSC2 expression levels under treatment with different drugs in the same cell. **f** p-TSC2 expression levels in *TSC2*-NC, WT, and MT groups treated with the same drugs. **g** p-mTOR expression levels under treatment with different drugs in the same cell. **h** p-mTOR expression levels in *TSC2*-NC, WT, and MT groups treated with the same drugs. **i** p-P70S6K expression levels under treatment with different drugs in the same cell. **j** p-P70S6K expression levels in *TSC2*-NC, WT, and MT groups treated with the same drugs. Data are shown as mean ± SD; One-way ANOVA was used to analyze the data in (**d**, **f**, **h**, **j**). Error bars are SEM. ^*^P < 0.05, ^**^P < 0.005, ^***^P < 0.0005, ^****^P < 0.0001.

We examined potential associated carcinogenic signaling pathways (Fig. 3a, b). In the untreated group, the total expression and phosphorylation levels of HER2 in the *TSC2*-MT group were significantly lower than those in the *TSC2-*NC and *TSC2-*WT groups (*n* = 3; F = 11.09, *P* = 0.0097; one-way ANOVA; Fig. 3c, d). Under conditions of lapatinib treatment, the phosphorylation levels of HER2 in the NC, WT, and MT groups were all significantly lower than those without treatment. Palbociclib treatment increased the phosphorylation of EGFR and HER2 in all three groups, particularly the phosphorylation of HER2 in the MT group (*n* = 3; F = 13.13, *P* = 0.0064; one-way ANOVA; Fig. 3d). The inhibition of HER2 became more pronounced after combination treatment. Western blotting results showed that in the untreated group, Thr1462 phosphorylation of *TSC2* was higher in the MT group than in the other two groups (*n* = 3; F = 8.982, *P* = 0.016; one-way ANOVA; Fig. 3e, f). Phosphorylation of mTOR and P70S6K was also significantly higher in the MT group than in the NC and WT groups (*n* = 3; F = 5.638, *P* = 0.0419 and F = 9.897, *P* = 0.0126, respectively; one-way ANOVA; Fig. 3h, i, j). Regarding the *TSC2* mutation, the phosphorylation levels of TSC2 and mTOR in the MT group were not inhibited by lapatinib, although they were inhibited in the NC and WT groups (*n* = 3; F = 244.1, *P* < 0.0001 and F = 37.06, *P* = 0.0004, respectively; one-way ANOVA; Fig. 3g, h). These results show that the *TSC2* c.4349 C > G (p.Pro1450Arg) variant upregulated signaling pathways and enhanced lapatinib resistance with an increase in the phosphorylation level of TSC2.

Next, we sought to determine how palbociclib suppressed P70S6K activity and relieved feedback inhibition on EGFR and HER2 in the MT group. Cyclin D1/CDK4 binds to and regulates the phosphorylation level of TSC2^13^, and palbociclib is a CDK4/6 inhibitor. In the MT group, the levels of p-TSC2, p-mTOR, and p-P70S6K were significantly lower than those in the NC and WT groups under palbociclib treatment (*n* = 3; F = 32.23, *P* = 0.001, F = 24.52, *P* = 0.0013, and F = 32.23, *P* = 0.0006, respectively; one-way ANOVA; Fig. 3f, h, j). Increased P70S6K activity is known to enhance the feedback inhibition of EGFR family kinase signaling^13^. After treatment with palbociclib, the phosphorylation levels of HER2 in the MT group were higher than those in the other two groups (Fig. 3d).

After lapatinib treatment, the expression of CDK4 and p-Rb was higher in the MT group than in the other two groups (Fig. 3b). Palbociclib was able to downregulate the expression of CDK4 and p-Rb in all three groups (Fig. 3b). The lapatinib + palbociclib combination completely inhibited the phosphorylation of the proteins involved in the TSC2, EGFR, HER2, and Rb pathways, with no significant differences observed among the three groups. Thus, palbociclib may have enhanced the effect of lapatinib on cells by inhibiting CDK4/6 and TSC2/mTOR signaling pathways.

### Lapatinib and palbociclib suppress the growth of HER2-positive breast cancer cells in xenograft models

**Fig. 4:**
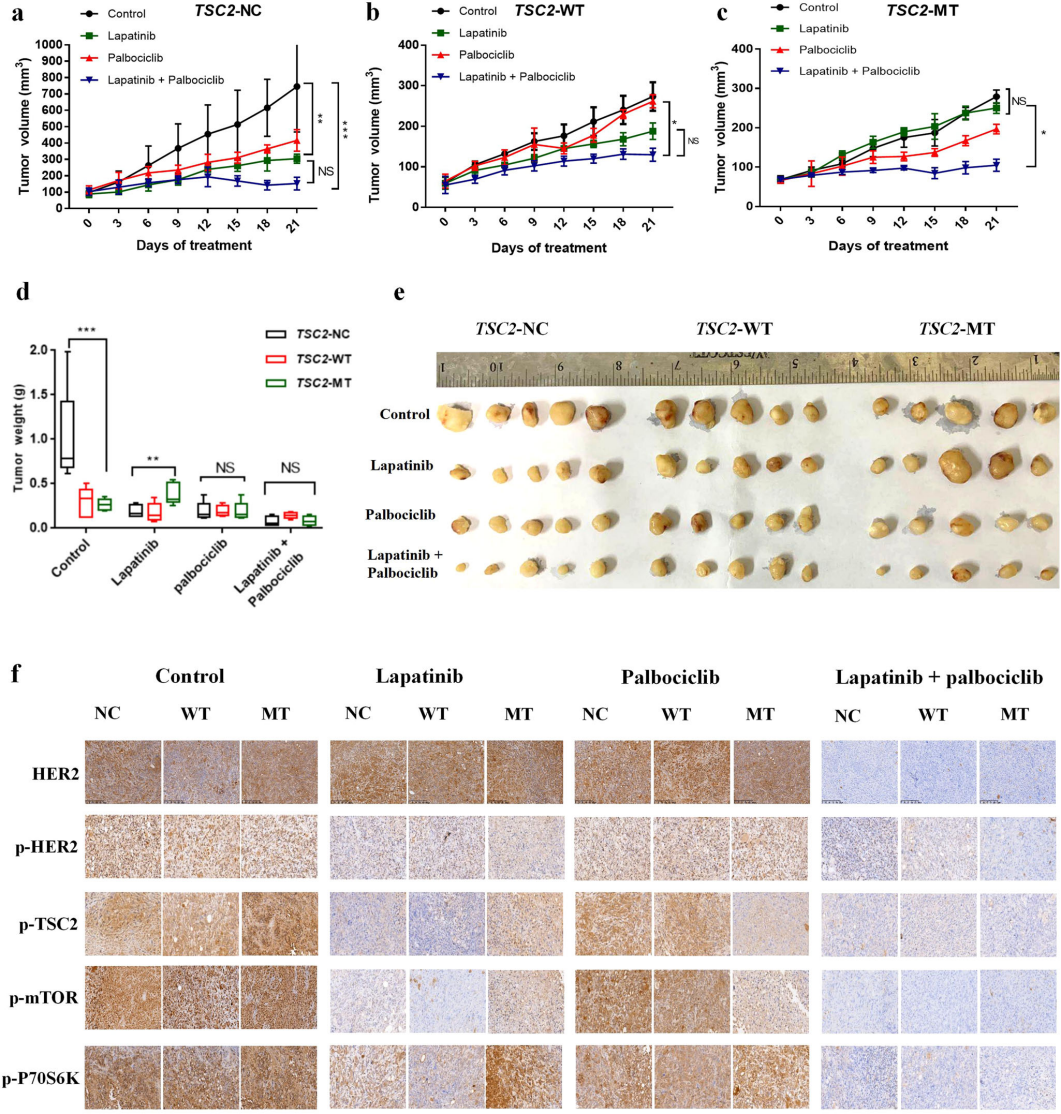
Tumor volume and weight in mice harboring BT474 cell lines treated with lapatinib and/or palbociclib. **a, b**, and **c** Cells were injected subcutaneously into nude mice and continuously observed for 21 days. Tumor-bearing mice were randomly divided into four groups: control, lapatinib treatment (80 mg/kg/d), palbociclib treatment (100 mg/kg/d), and combination treatment (80 mg/kg/d + 100 mg/kg/d) for 21 days. Relationship between in vivo tumor growth rate and days after drug treatment for *TSC2*-NC, *TSC2*-WT, and *TSC2*-MT cells. Tumor volumes were measured every 3 days after drug treatment. **d** After day 21 treatment, the tumors from the control, lapatinib, palbociclib, and combination groups were dissected and weighed. Box plots indicate median and interquartile range. The lower and upper hinges correspond to the first (25th percentile) and third (75th percentile) quartiles, respectively. **e** Images of the tumors captured after 21 days of continuous drug administration. The first tumor of the *TSC2*-NC group in the control was too large, so only half of it is shown in the figure for representative purposes. Scale bar for each image is 1 cm. **f** Representative IHC results showing HER2, p-HER2, p-TSC2, p-mTOR, and p-P70S6K expression in BT474 tumors after different drug treatments in the xenograft models. IHC images in **(f)** are at ×20 magnification, scale bar: 100 μm. Data are shown as mean ± SD; one-way ANOVA was used to analyze the data in (**a**, **b**, **c**, **d**). Error bars are SEM. ^**^P < 0.05, ^***^P < 0.01.

Based on the tumor growth curve, the increase in tumor volume in the *TSC2*-NC group was significantly different in the control and lapatinib groups (F = 8.187, *P* = 0.0005; one-way ANOVA). A significant difference was also observed between the control and combination therapy groups (F = 22.33, *P* = 0.0001; one-way ANOVA; Fig. 4a). Moreover, in the *TSC2*-WT group, a significant difference was observed in the tumor volume between the palbociclib and combination treatments (F = 1.809, *P* = 0.001; one-way ANOVA); however, combination therapy showed no significant inhibitory effect over lapatinib monotherapy in this group (F = 2.529, *P* = 0.0776; one-way ANOVA; Fig. 4b). In the *TSC2*-MT group, no significant difference was observed between the growth curves for the lapatinib and control treatments, indicating that lapatinib had no significant inhibitory effect on tumor growth in this group. However, when compared with the lapatinib group, the combination group showed significantly inhibited tumor growth (F = 3.531, *P* = 0.0275; one-way ANOVA Fig. 4c). The combination of lapatinib and palbociclib exerted a stronger inhibitory effect on the growth of xenografts than either drug alone.

In the absence of treatment, tumor growth in the NC group was significantly higher than that in the other two groups (NC = 0.99 ± 0.56 g, WT = 0.29 ± 0.07 g, and MT = 0.26 ± 0.06 g; *n* = 3, F = 7.60, *P* = 0.0074; one-way ANOVA). With lapatinib alone, tumor weight in the MT (0.385 ± 0.121 g) group was significantly higher than that in the NC and WT groups (NC: 0.195 ± 0.07 g, WT: 0.177 ± 0.108 g; *n* = 3, F = 6.45, *P* = 0.0125; one-way ANOVA). These results showed that for the MT group, tumor weight was significantly lower in mice injected with a combination of the two drugs (0.086 ± 0.052) than in mice injected with lapatinib alone (0.385 ± 0.121, *n* = 2, t = 5.09, *P* = 0.0009; T test). In the case of combination treatment, no significant difference was observed in tumor weight among the three groups (*n* = 3, F = 0.462, *P* = 0.147; one-way ANOVA). In summary, the in vivo experiment results revealed that the MT group showed resistance to lapatinib. The combination of lapatinib and palbociclib was shown to markedly inhibit the relative growth of tumors and enhance anticancer activity, compared with lapatinib monotherapy (Fig. 4d).

### Immunohistochemistry

We verified that the two drugs (either alone or in combination) inhibited tumor growth in a HER2-amplified breast cancer xenograft mouse model. Given that the first tumor of the *TSC2*-NC control group treatment was too large, the image of half of the tumor is presented in Fig. 4e. To support these results, we performed immunohistochemical (IHC) analysis using mouse tumor tissues (Fig. 4f). In three groups of xenografts, lapatinib significantly inhibited the phosphorylation of HER2, while palbociclib increased the phosphorylation of HER2. The lapatinib-treated tumor tissues showed reduced expression of p-mTOR, p-TSC2, and p-P70S6K in the *TSC*-NC and *TSC2-*WT groups. However, the expression of these proteins was not significantly reduced in the *TSC2-*MT group. The combination of lapatinib and palbociclib resulted in a greater reduction in the phosphorylation of TSC2, mTOR, and P70S6K. These results are consistent with those obtained at the cellular level and confirm the benefits of the combined approach targeting CDK4/6 and HER2 and their downstream pathways.

## Discussion

**Fig. 5:**
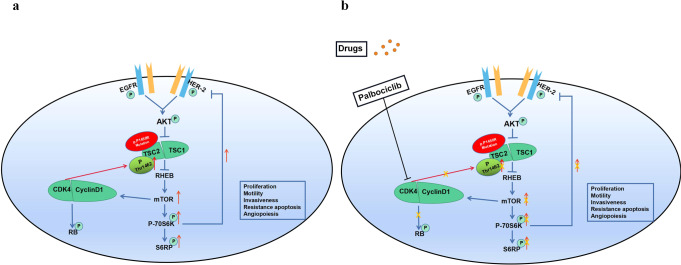
Palbociclib reverses anti-HER2 resistance induced by the *TSC2* c.4349 C > G (p.Pro1450Arg) variant. **a** Phosphorylation level of TSC2 p.Pro1450Arg mutant on Thr1462 increased, which led to activation of the mTOR pathway. In addition, phosphorylation levels of mTOR and P70S6K increased. P70S6K partially suppresses the RTK signal of EGFR family members via negative feedback. The potential mechanism of increased phosphorylation in the TSC2 p.Pro1450Arg variant on Thr1462 might be caused by cyclin D1/CDK4 binding to and regulating the phosphorylation level of TSC2. **b** After palbociclib treatment, the activation of CDK4 was inhibited, leading to decreased phosphorylation of TSC2, mTOR, and P70S6K. As a result, the feedback inhibition of the EGFR family by P70S6K was attenuated. Moreover, palbociclib effectively inhibited Rb phosphorylation, resulting in some cells stagnating in the G1 phase. (p: phosphorylation).

Approximately 6000 variants of *TSC2* are registered in ClinVar (www.ncbi.nlm.nih.gov/ClinVar/), including over 700 pathogenic or likely pathogenic mutations leading to TSC. *TSC2* c.4349 C > G (p.Pro1450Arg) was classified as a variant demonstrating conflicting interpretations of pathogenicity. This variant has been mentioned in previous studies; however, its function has not been analyzed and its clinical significance remains unknown^14^. The HER2-positive breast cancer patient harboring the *TSC2* germline nonsynonymous variant c.4349 C > G was resistant to trastuzumab administered during neoadjuvant therapy. The present study found that this variant promoted the phosphorylation of Thr1462 in TSC2, which enhanced the activation of mTOR and its downstream signals and increased the number of cells in the G2 + M + S phase of the cell cycle, leading to the development of resistance to lapatinib (Fig. 5a). However, palbociclib also inhibited the cyclinD1/CDK4-Rb alternative pathway, indirectly reducing the phosphorylation of Thr1462 in TSC2 by inhibiting the binding between CDK4 and TSC2 (Fig. 5b). A previous study found that pathogenic mutations in *TSC2* and *TSC1* disrupt the functional complex formed by the protein products of these two genes that influence mTOR signaling, leading to constitutive mTORC1 activation^15^. The in vivo targeting effect of rapamycin can eliminate *TSC2*-null xenograft tumor model growth, induce apoptosis, increase the survival rate of tumor-bearing mice, and prevent tumor regeneration after treatment^16^. Thus, the *TSC2* c.4349 C > G variant was found to be related to anti-HER2 treatment resistance.

Our results showed that the combination of lapatinib and palbociclib could block the cell cycle in the G1 phase and that palbociclib could re-sensitize *TSC2*-mutant cells to anti-HER2 therapy. Zhang et al.^17^ also found that a combination of palbociclib and pyrotinib induced G0/G1 cell cycle arrest and strongly inhibited tumor cell proliferation and colony formation. In this study, the combined treatment significantly reduced p-mTOR activation and increased tumor cell apoptosis. Goels et al.^18^ revealed that dual inhibition of HER2 and CDK4/6 induces a highly potent suppression of TSC2 phosphorylation, thereby markedly blocking mTORC1/S6K/S6RP activity. Consequently, CDK4/6 inhibition could restore the sensitivity of drug-resistant tumor cells to the effects of HER2 inhibitors. These studies demonstrate a possible mechanism underlying these synergistic effects. The cyclinD1/CDK4 pathway promotes cell proliferation via phosphorylation of Rb^19^. Zacharek et al.^13^ reported that cyclinD1/CDK4 could bind to TSC2 and upregulate its phosphorylation, thereby negatively affecting the stability of the TSC1/TSC2 complex. A related study^20^ has shown that the expression of wild-type *TSC*2 could reduce mTORC1 activity, which is further downregulated after treatment with CDK4 inhibitors. In our study, inhibition of CDK4/6 led to the downregulation of mTOR signaling with a reduction in the phosphorylation of the Thr1462 residue of TSC2 in *TSC2*-mutant cells. As expected, the phosphorylation levels of both mTOR and P70S6K were decreased, indicating that CDK4/6 inhibitors could inhibit the binding of CDK4 and TSC2. This led to a reduction in the phosphorylation of TSC2 (p1462) and inhibition of the mTOR pathway^11^.

In our study, the lapatinib + palbociclib combination treatment, targeting the HER2/AKT/mTOR signaling pathway and inhibiting the CDK4/6-Rb bypass pathway, significantly inhibited the survival of tumor cells and induced apoptosis. These findings were also supported by the results of the xenograft model experiments. A recent study^21^ demonstrated that dysregulation of the CCND1–CDK4/6‐Rb axis contributes to pyrotinib resistance, and that CDK4/6 inhibitor (SHR6390) sensitizes pyrotinib in a HER2‐positive advanced gastric cancer mouse model. Our data confirmed the sensitizing effect of a CDK4/6 inhibitor on lapatinib and suggested mechanisms by which lapatinib and palbociclib synergistically work against HER2 therapy-resistant breast cancer in a nude mouse model. The combination of a CDK4/6 inhibitor and anti-HER2 treatment has demonstrated a high response rate and is well-tolerated in patients.

Our study has shortcomings that should be noted. We used lapatinib, and not trastuzumab, to investigate the resistance of anti-HER2 treatment in the BT474 cell line overexpressing the *TSC2* mutant, as trastuzumab and lapatinib can both inhibit HER2. Additionally, lapatinib can inhibit HER1, and trastuzumab induces apoptosis of cancer cells by blocking the HER2 signal pathway and the antibody-dependent cell-mediated cytotoxicity effect, which could lead to different anti-cancer effects and mechanisms of resistance. The patient carrying the *TSC2* c.4349 C > G variant was resistant to trastuzumab in neoadjuvant treatment, making it more meaningful to test trastuzumab rather than lapatinib in this study. We also employed two-dimensional (2D) culture in this study, which could impose some limitations. Cells grown in a 2D culture environment are generally flatter and more extended than those grown in vivo. Moreover, the abnormal cell morphology in 2D cultures affects cell proliferation, differentiation, apoptosis, and gene expression. These limitations could be avoided by using three-dimensional (3D) culture, which is capable of modeling normal cell growth in vivo. Pickl et al.^22^ found that a 3D culture system might better reflect HER signaling than 2D culture. Rodríguez et al.^23^ suggested that 3D architecture could modify breast cancer cell response to trastuzumab by modulating stem cell population and HER2 distribution. Tatara et al.^24^ reported increased expression of cleaved PARP and a decreased ratio of p-AKT to AKT in 3D-cultured *PIK3CA*-WT HER2-positive cell lines in response to trastuzumab, but not in 2D-cultured *PIK3CA*-WT lines. As 3D culture could better reflect HER signaling conditions and trastuzumab action in vivo, it will be employed in future studies to further assess the trastuzumab resistance mechanism in *TSC2*-MT and HER2-positive breast cancer cell lines.

To the best of our knowledge, this is the first study to identify that the *TSC2* (NM_000548) c.4349 C > G (p.Pro1450Arg) variant could activate the HER2/AKT/mTOR pathway. This variant is related to anti-HER2 resistance and could be controlled by CDK4/6 inhibition. We demonstrate a new mechanism underlying anti-HER2 resistance and provide the rationale for using a combination of anti-HER2 and CDK4/6 inhibitors in HER2-positive breast cancer.

## Methods

### Patient and next-generation sequencing (NGS) analysis

Written informed consent was obtained from the patient for the use of samples and information, as well as for the publication of results. This study was conducted in accordance with the Declaration of Helsinki and was approved by the Research and Ethics Committee of the Zhejiang Cancer Hospital, China (IRB-2017-199).

A 33-year-old female patient was diagnosed with stage IIIA (cT3N1M0) breast cancer in August 2016. IHC demonstrated the following results: HER2 (3+), ER (+, 20%), PR (−), and Ki-67 (+, 70%). The molecular type was luminal B (HER2 positive). From August 22, 2016, to January 10, 2017, the patient underwent neoadjuvant treatment as follows: AC*4 regimen (doxorubicin+cyclophosphamide) followed by PH*4 regimen (paclitaxel+trastuzumab). Treatment efficacy was evaluated according to the Response Evaluation Criteria in Solid Tumors version 1.1 guidelines^25^.

Genomic DNA was prepared using a QIAamp DNA Blood Mini kit (QIAGEN, catalog number 51104, Hamburg, Germany) from peripheral blood samples. A 98-gene panel sequencing assay was employed to analyze the germline mutations in HiSeq X-Ten (Illumina, catalog number 56404, San Diego, CA, USA), as the patient was diagnosed with early-onset breast cancer for hereditary screening (the 98 genes are listed in Supplementary Table 1). The total DNA was extracted using an FFPE DNA extraction kit (QIAGEN, catalog number 51104). Whole-exome capture was performed using SureSelect Human All Exon V6 Kit (Agilent Technologies, catalog number 5190-8863, Santa Clara, CA, USA). Sequencing was performed using the Illumina Novaseq 6000. Somatic SNVs and InDels were jointly called by Mutect2^26^. Annovar was utilized to add biological information to a set of variants (https://annovar.openbioinformatics.org/en/latest/).

### Cell culture

The breast cancer cell lines BT474 (NICR, Procell, catalog number CL-0040) and HEK293T (NICR, Procell, catalog number CL-0005) were purchased from the Chinese National Infrastructure of Cell Line Resource (NICR) and tested for mycoplasma contamination. The cells were cultured in Roswell Park Memorial Institute (RPMI)-1640 medium (Jinuo Biomedical Technology, Hangzhou, China) supplemented with 10% fetal bovine serum, 100 U/mL penicillin (Invitrogen, Thermo Fisher Scientific, Waltham, MA, USA), and 100 mg/mL streptomycin (Jinuo Biomedical). The cells were grown at 37 °C in an atmosphere containing 5% carbon dioxide with saturated humidity. Only cells in the logarithmic growth phase were digested for subcutaneous inoculation.

### Overexpression of mutant *TSC2*

The cells were divided into three groups, i.e., negative control (NC), wild-type *TSC2* overexpression (*TSC2*-WT), and mutant *TSC2* overexpression (*TSC2*-MT). The *TSC2*-WT sequence was amplified by polymerase chain reaction at the restriction site (BamHI/AgeI) and recombined into the vector GV492_Ubi-MCS-3FLAG-CBh-gcGFP-IRES-puromycin. (catalogue number GV492). The variant site of *TSC2* was c.4349 C > G (p.Pro1450Arg), and the corresponding specific primers are as follows: F, 5’-CGCTATGTGGATACGCTGCTTTA-3’ and R, 5’-GCAACCAGGATTTATACAAGGAGGA-3’. Similarly, *TSC2*-MT was amplified at the BamHI/AgeI site and recombined into the vector GV492_Ubi-MCS-3FLAG-CBh-gcGFP-IRES-puromycin. The recombinant construct was verified by sequencing and then packaged with lentivirus. The virus was collected after 48 h to determine the concentration and titer. This lentivirus was used to transduct 293 T cells, and transduction efficiency was determined under a fluorescence microscope. After screening with puromycin (1 µg/mL), the expression of the target protein was determined via Western blotting.

### Western blotting

The cells were collected and homogenized in a lysis buffer supplemented with protease inhibitor (Beyotime, Shanghai, China) and phenylmethylsulfonyl fluoride (1 mM, Beyotime, Shanghai, China) for 30 min on ice. The lysates were centrifuged at 12000 *g* at 4 °C for 10 min and supernatants were collected. Before blotting, protein concentration was measured using the bicinchoninic acid protein assay (HyClone-Pierce, Rockford, IL, USA). For each group, protein samples were separated using 10% sodium dodecyl sulfate-polyacrylamide gel electrophoresis and transferred onto polyvinylidene fluoride membranes. The membranes were first blocked in milk and then probed with different antibodies, such as rabbit anti-TSC2 (catalog number 24601-1-AP, 1:1000 dilution, Proteintech, Rosemont, IL, USA), rabbit anti-mTOR (catalog number 28273-1-AP, 1:1000 dilution, Proteintech), rabbit anti-P70S6K (catalog number 14485-1-AP, 1:1000 dilution, Proteintech), mouse anti-GAPDH (catalog number 60004-1-Ig, 1:2000 dilution, Proteintech) and mouse anti-actin (catalog number 66009-1-Ig, 1:2000 dilution, Proteintech) antibodies. Additionally, rabbit anti-p-mTOR (Ser2448, catalog number 5536, 1:1000 dilution, Cell Signaling Technology Inc., Danvers, MA, USA), rabbit anti-p-P70S6K (Thr389, catalog number 9234, 1:1000 dilution, Cell Signaling Technology), rabbit anti-CDK4 (catalog number 12790, 1:1000 dilution, Cell Signaling Technology), mouse anti-Rb (catalog number 9309, 1:1000 dilution, Cell Signaling Technology), rabbit anti-p-Rb (Ser807/811, catalog number 8516, 1:1000 dilution, Cell Signaling Technology) as well as rabbit anti-p-TSC2 (Thr1462, catalog number AF3334, 1:1000 dilution, Affinity Biosciences, Cincinnati, OH, USA), rabbit anti-HER2 (catalog number AF7681, 1:1000 dilution, Affinity Biosciences), rabbit anti-p-HER2/ERBB2 (Tyr1248, catalog number AF3069, 1:1000 dilution, Affinity Biosciences), rabbit anti-EGFR (catalog number AF6043, 1:1000 dilution, Affinity Biosciences), and anti-p-HER1/EGFR (Tyr1173, catalog number AF3048, 1:1000 dilution, Affinity Biosciences) antibodies were used. After washing thrice with Tris-buffered saline and Tween, the membranes were incubated with the corresponding secondary antibodies (goat anti-mouse or goat anti-rabbit antibodies; Proteintech). The bands were visualized using an enhanced chemiluminescence kit (Beyotime).

### Flow cytometry

*TSC2*-NC, *TSC2-*WT, and *TSC2-*MT cells were suspended in a flow buffer which was phosphate-buffered saline (PBS) containing 2% fetal bovine serum and were fixed with paraformaldehyde for 10 min; the membrane was then broken with a membrane-breaking solution for 20 min. After a subsequent wash in the flow PBS, cells were centrifuged at 3000 × *g* and again suspended in PBS and incubated with anti-EGFR (CB2 0AX, Abcam, Cambridge, UK) and anti-HER2 antibodies (Cell Signaling Technology) in the dark for 2 h at 4 °C, with a subsequent wash in the flow buffer, centrifuged at 3000 *g* and again suspended in PBS. The cells were stained with the secondary antibodies Alexa Fluro 405 anti-rabbit IgG (ab175651, Abcam, Cambridge, UK) and Alexa Fluor 594 anti-mouse (ab150108, Abcam) in the dark for 30 min at 4 °C. After staining, the cells were analyzed using flow cytometry (BD CantoII, Becton Dickinson, San Jose, CA, USA).

### Cell viability assessment

Cell viability was determined using Cell Counting Kit 8 (CCK8; Dojindo, Tokyo, Japan), according to the manufacturer’s guidelines, to determine the IC_50_ values of different treatments. First, the different groups of BT474 cells were seeded in 96-well plates at a density of 5 × 10^4^ cells/well as six replicates in 0.2 mL of the culture medium; medium containing no cells was used as control. After attachment, the cells were incubated with different concentrations of lapatinib (1.5–25 µM) and palbociclib (1–30 µM) for 48 h. CCK8 reagent was added; after 2 h, the optical density (OD) was measured at 450 nm (Bio-Rad, Hercules, CA, USA), and the IC_50_ of the drug was calculated based on the OD value. Cell cytotoxicity was calculated according to the following formula: inhibition ratio (%) = (OD (drug − OD (blank))/(OD (drug control) − OD (blank)) × 100%. Finally, using the same method as above, lapatinib (1–30 µM) was added at a 20% inhibitory concentration (IC_20_) with palbociclib. The growth rate was evaluated in the same manner for 48 h by measuring absorbance at 450 nm. The CI values were calculated using CompuSyn (ComboSyn Inc., New York, NY, USA)^12^. The fraction affected was determined according to the following formula: 100 – growth inhibition/100. The CI value was used to determine whether the effects of the two drugs were synergistic (<1), additive (1–1.2), or antagonistic (>1.2) (Table 1).

**Table 1:** Recommended symbol table for describing synergy or antagonism in drug combination studies analyzed by the combination index (CI) method.

Range of CI	Symbol	Description
<0.1	+++++	Very strong synergism
0.1–0.3	++++	Strong synergism
0.3–0.7	+++	Synergism
0.7–0.85	++	Moderate synergism
0.85–0.90	+	Slight synergism
0.90–1.10	±	Nearly additive
1.10–1.20	–	Slight antagonism
1.20–1.45	– –	Moderate antagonism
1.45–3.3	– – –	Antagonism
3.3–10	– – – –	Strong antagonism
>10	– – – – –	Very strong antagonism

### Cell cycle analysis

The three groups of cells in the logarithmic growth phase were seeded in a 6-well plate (10^5^ cells/well) and were starved for 24 h before processing. The different groups of BT474 cells were treated with lapatinib, palbociclib, or a combination of the two drugs for 48 h. The cells were washed with cold PBS and treated with 70% ethanol at 4 °C overnight, and the cell solution was adjusted to obtain 5 × 10^5^ cells/mL. The cells were then stained with propidium iodide (50 µg/mL, MultiSciences, Hangzhou, China) in the presence of RNase A (100 µg/mL). A flow cytometer (Beckman Coulter flow cytometer, Brea, CA, USA) was used to evaluate cell cycle distribution. Each experiment was performed in triplicate.

### Colony formation assay

The three groups of cells (*TSC2*-NC, *TSC2*-WT, and *TSC2*-MT) were seeded in 6-well plates at a density of 500 cells/well and treated with different concentrations of lapatinib, palbociclib, or a combination of the two drugs for 2 days. The cells were cultured for an additional 2 weeks. Colonies (>50 cells) were stained with crystal violet, and each well was photographed before the colonies were counted under an inverted microscope.

### Xenograft studies

This study was approved by the Laboratory Animal Management and Ethics Committee of Zhejiang University of Traditional Chinese Medicine, China (20190708-08). Female nude mice (BALB/c: female thymus free, age: 4–6 weeks, weight: approximately 30 grams) were purchased from the Shanghai Slack laboratory Animal Co., Ltd. (Shanghai, China) and raised in a pathogen-free animal facility at the Zhejiang University of Traditional Chinese Medicine. The investigators showed no bias in the processing of animal experiments. Experiments complied with all relevant ethical regulations for animal testing and research. The sample size of mice was determined using the degree of freedom (E) of variance analysis, and E was calculated as follows: total number of mice in each group − total number of groups. The range of E was 10–20. A total of 60 mice were used. The laboratory technician randomly divided the mice into *TSC2*-NC, *TSC2*-WT, and *TSC2*-MT groups (20 mice/group) using the simple randomization method with a random number table. Three groups of *TSC2*-NC, *TSC2*-WT, and *TSC2*-MT xenografts were suspended in 1:4 matrix gel at 10^7^ cells/100 μL and injected subcutaneously into the right side of the dorsal surface of each mouse. As the tumor formation rates were 100% in all three groups, no mice were excluded. When the tumor volume was close to 100 mm^3^, all mice in each group were randomly divided into control, lapatinib, palbociclib, and combination of two drugs groups (5 mice/group/cage) using the same randomization method. Group 1, the control group, received vehicle (0.5% methylcellulose, 0.4% Tween-80); group 2 received lapatinib (80 mg/kg/d); group 3 received palbociclib (100 mg/kg/d); and group 4 received lapatinib (80 mg/kg/d) and palbociclib (100 mg/kg/d). As all drugs are water-soluble and absorbed through the intestine, they were administered once daily via oral gavage at the same time for 21 consecutive days. All mice were maintained under the same controlled feeding and housing conditions. Tumors were measured twice/week using a caliper in the same order every time, and their volumes (mm^3^) were calculated using the formula (width)^2^ × length/2. After 21 days, mice were sacrificed by cervical dislocation without anesthesia, and tumors were harvested after the last treatment for tumor weight measurement. Tumor tissues were preserved in liquid nitrogen, or formalin-fixed and paraffin embedded (FFPE) for analysis of associated biomarkers (HER2, p-HER2, p-TSC2, mTOR, and p-P70S6K).

### Immunohistochemistry

We performed IHC to evaluate the clinical and animal tumor samples. Paraffin sections with a thickness of 5 µm were deparaffinized with xylene and absolute ethanol. Antigen recovery was performed after washing with distilled water. The paraffin sections were incubated with 3% hydrogen peroxide at room temperature (22 °C) for half an hour and blocked with bovine serum albumin for the same duration followed by overnight probing with the primary antibody. anti-HER2 (catalog number AF7681, 1:100 dilution, Affinity Biosciences), rabbit anti-p-HER2/ERBB2 (Tyr1248, catalog number AF3069, 1:100 dilution, Affinity Biosciences). anti-p-TSC2 (Thr1462, catalog number AF3334, 1:50 dilution, Affinity Biosciences). anti-p-mTOR (Ser2448, catalog number 5536, 1:100 dilution, Cell Signaling Technology), rabbit anti-p-P70S6K (Thr389, catalog number AF3228, 1:100 dilution, Affinity Biosciences). Finally, the sections were washed thrice with PBS. The sample was then incubated with horseradish peroxidase-linked secondary antibody from the immunohistochemistry kit (Dako, catalog number K5007, Copenhagen, Denmark). After incubating for 1 h and washing thrice, diaminobenzidine solution was added. The sections were then stained and counterstained with hematoxylin, dehydrated, and observed under a fluorescence microscope.

### Statistical analyses

Continuous quantitative data were compared using the independent-sample Student’s *t*-test (between two groups) or one-way ANOVA (more than two groups). Data analyses were performed using GraphPad Prism (GraphPad Software Inc., La Jolla, CA, USA), and data were presented as mean ± standard deviation. For all data, *P* < 0.05 was considered significant.

### Reporting summary

Further information on research design is available in the Nature Research Reporting Summary linked to this article.

## Supplementary information

Supplementary Material

Reporting Summary

## Data Availability

The raw data of whole-exome sequencing from the breast cancer patient with *TSC2* c.4349 C > G variant can be accessed here: https://www.ncbi.nlm.nih.gov/sra/PRJNA924228. Other data generated or analyzed during this study are included in this published article (and its supplementary information files). The complete version of the data is accessed from the corresponding author on reasonable request.

## Code availability

All statistical analyses were performed using SPSS version 15 or GraphPad Prism v7.0. Open-source software was employed for bioinformatic analyses in this study.
