# Supplementary material for: Resistance to anti-HER2 therapy associated with the TSC2 nonsynonymous variant c.4349 C > G (p.Pro1450Arg) is reversed by CDK4/6 inhibitor in HER2-positive breast cancer
Source: NPJ Breast Cancer. 2023 May 9;9:36. doi: 10.1038/s41523-023-00542-1 (PMC10170158; doi:10.1038/s41523-023-00542-1)

Supplementary Table 1. List of 98 inherited cancer genes

Cancer	Gene
Breast cancer	*ATM*, *BARD1*, *BLM*, *BRCA1*, *BRCA2*, *CDH1*, *CHEK2*, *FANCC*, *FANCM*, *MCPH1, MEN1*, *MLH1*, *MRE11a*, *MSH2*, *MSH6*, *MUTYH*, *NBN*, *NF1*, *PALB2*, *PMS2*, *PTEN*, *RAD50*, *RAD51C*, *RAD51D*, *STK11*, *TP53*, *XRCC2*
Ovarian cancer	*BARD1, BRCA1*, *BRCA2*, *BRIP1*, *CASR*, *EPCAM*, *FAM175A*, *MLH1*, *MRE11A*, *MSH2*, *MSH6*, *NBN*, *PALB2, PMS2*, *RAD50*, *RAD51C*, *RAD51D*, *STK11*
Lung cancer	*BAP1*, *EGFR*
Stomach cancer	*BLM*, *CDH1*, *EPCAM*, *KIT*, *MLH1*, *MSH2*, *MSH6*, *PDGFRA*, *PMS2*, *SDHA*, *STK11*
Liver cancer	*GPC3*
Esophageal carcinoma	*APC*, *MLH1*
Colo-Rectal Carcinoma	*AXIN2*, *APC*, *BLM*, *BMPR1A*, *CASR*, *CDH1*, *CHEK2, GREM1*, *KIT*, *MLH1*, *MSH2*, *MSH6*, *MUTYH*, *PDGFRA*, *PMS1*, *PMS2*, *PTEN*, *SDHA*, *SMAD4*, *POLD1*, *POLE*, *STK11*
Prostate cancer	*BRCA1*, *BRCA2*, *CHEK2*, *HOXB13*, *NBN*
Bladder Cancer	*HRAS*
Pancreatic cancer	*ATM*, *BRCA1*, *BRCA2*, *CDKN2A*, *EPCAM, MLH1*, *MSH2*, *MSH6*, *PALB2*, *PMS2*, *STK11*
Lymphoma	*ATM*, *BLM*, *NBN*, *PAX5, PRF1*
Cerebroma	*BAP1*, *MLH1*, *MSH2*, *MSH6*, *NF2*, *PMS2*, *SMARCE1*
Thyroid Carcinoma	*CDC73*, *MEN1*, *PRKAR1A*, *PTEN*, *RET*, *WRN*
Cervical cancer	*STK11*
Endometrium carcinoma	*EPCAM*, *MLH1*, *MSH2*, *MSH6*, *PMS2*, *PTEN*
Others	*AIP*, *ALK*, *CDK4*, *CDKN1B*, *CDKN1C*, *CEBPA*, *DICER1*, *DIS3L2*, *FANCA*, *FANCB*, *FANCD2*, *FANCE*, *FANCF*, *FANCG/XRCC9*, *FANCI*, *FANCL*, *FANCP/SLX4*, *FANCQ/ERCC4*, *FANCR/RAD51*, *FANCT/UBE2T, FH*, *FLCN*, *GATA2*, *MAX*, *MET*, *PHOX2B*, *PTCH1*, *RB1*, *RECQL*, *RECQL4*, *RUNX1*, *SMARCA4*, *SDHAF2*, *SDHB*, *SDHC*, *SDHD*, *SMARCB1*, *SUFU*, *TMEM127*, *TERC*, *TSC1*, *TSC2*, *VHL*, *WT1*

Supplementary Table 2. Somatic alternations identified in the breast cancer patient with *TSC2* c.4349C>G germline variant

Chr	Gene	AA Change	Function	SIFT	Polyphen-2
chr1	UBR4	NM_020765:exon59:c.8609_8610insGTGCAGTGGACTCAGTGGCTGG:p.D2870Efs*26	frameshift insertion	.	.
chr1	FAF1	NM_007051:exon10:c.884_885insTTTGGCATGGCGTCAT:p.F296Lfs*7	frameshift insertion	.	.
chr1	KRTCAP2	NM_173852:exon3:c.238delG:p.A80Pfs*94	frameshift deletion	.	.
chr1	OR10K1	NM_001004473:exon1:c.553_554insTTACCCCATTGTTCAATCCAATGATTTATAGTCTGAGAAATAAGGAATTCAAATCAGCCCTACGAAGAACAATCGGCCAAACTTTCTATCCTCTTAG:p.K186Tfs*33	frameshift insertion	.	.
chr1	IGFN1	NM_001164586:exon12:c.3769_3823del:p.G1258Vfs*35	frameshift deletion	.	.
chr2	RANBP2	NM_006267:exon20:c.G7083C:p.Q2361H	nonsynonymous SNV	Damaging	Possibly damaging
chr2	NEB	NM_004543:exon72:c.10472_10473insTTTTTTTTCTTTGCCTTTTA:p.D3492_I6657delinsFFFFAF*	stopgain	.	.
chr2	COQ10B	NM_001320818:exon4:c.452_453insCTTTCTTTCCTTCTTTCTTTCTCTCTCTC:p.D152Ffs*14	frameshift insertion	.	.
chr3	CD200	NM_001318828:exon3:c.302_303insAGTACAGCCCATAGTATCCCTTCACTACAAAT:p.R102Vfs*20	frameshift insertion	.	.
chr3	ACTL6A	NM_004301:exon13:c.1180_1184del:p.I395Rfs*21	frameshift deletion	.	.
chr4	THEGL	NM_001256475:exon8:c.1135_1136insCTAGCCCCAGAACCCCAGCCTTTCCTGTCCCACTTTAGCCTGAAATTGACATACCTCTTATTTGGATTAG:p.I384Pfs*8	frameshift insertion	.	.
chr4	UGT2B28	NM_001207004:exon1:c.191_192insCAAGTCACTG:p.D65Kfs*4	frameshift insertion	.	.
chr4	HELQ	NM_001297755:exon2:c.818_819insACTGAGATTCTAATTTTTGA:p.M273Ifs*5	frameshift insertion	.	.
chr4	ENPEP	NM_001977:exon14:c.G2076A:p.W692X	stopgain	.	.
chr7	ZNF680	NM_178558:exon4:c.337_338insTGTGTGTGTGTGTGTGTGTTAGAGAGAGAGA:p.G113Vfs*16	frameshift insertion	.	.
chr7	SLC13A4	NM_001318192:exon13:c.1391_1392insTT:p.W465Sfs*34	frameshift insertion	.	.
chr7	SLC13A4	NM_001318192:exon13:c.1390_1391insTCTGG:p.P464Lfs*36	frameshift insertion	.	.

chr7	KMT2C	NM_170606:exon8:c.C1173A:p.C391X	stopgain	.	.
chr8	POP1	NM_001145860:exon6:c.760_761insCCTGTGTAAATTCCTA:p.E254Afs*6	frameshift insertion	.	.
chr8	SYBU	NM_001330596:exon5:c.G533C:p.R178P	nonsynonymous SNV	Damaging	Probably damaging
chr9	SPATA31D1	NM_001001670:exon4:c.405_421del:p.D139Vfs*23	frameshift deletion	.	.
chr9	TTC16	NM_001317037:exon6:c.492_493insCTTCCGTTACCGATGGTGA:p.A165Lfs*323	frameshift insertion	.	.
chr10	MKI67	NM_001145966:exon12:c.1932_1933insCGTTCTG:p.M645Rfs*67	frameshift insertion	.	.
chr11	PPP6R3	NM_001164163:exon17:c.C1822T:p.R608X	stopgain	.	.
chr11	ATM	NM_000051:exon23:c.3307_3308insACATAATAAC:p.S1104Ifs*21	frameshift insertion	.	.
chr12	NUP107	NM_001330192:exon23:c.G1919A:p.R640H	nonsynonymous SNV	Damaging	Probably damaging
chr12	CCT2	NM_001198842:exon7:c.465_508del:p.I155Mfs*6	frameshift deletion	.	.
chr16	TSC2	NM_000548:exon34:c.C4349G:p.P1450R	nonsynonymous SNV	Damaging	Probably damaging
chr16	RRN3	NM_001301064:exon15:c.1469_1470insAAAAAAAAAAAATCCACCTCCT:p.Y490*	frameshift insertion	.	.
chr16	REXO5	NM_001144924:exon3:c.C145T:p.R49C	nonsynonymous SNV	Damaging	Probably damaging
chr17	STARD3	NM_001165938:exon12:c.1037_1038insTCTTCTGCACTTTGGCCTTGGGTCATTGTCCCCT:p.I347Lfs*43	frameshift insertion	.	.
chr17	IKZF3	NM_001257408:exon4:c.362_477del:p.F121*	frameshift deletion	.	.
chr17	WIPF2	NM_133264:exon5:c.911_912insTGGG:p.S305Gfs*6	frameshift insertion	.	.
chr19	RYR1	NM_000540:exon38:c.6180_6181insAACACAAGGAAACCTCATTTCTACAAAAAATACAAAA:p.S2061Nfs*33	frameshift insertion	.	.
chr19	PSG2	NM_031246:exon4:c.C856G:p.L286V	nonsynonymous SNV	Damaging	Probably damaging
chr19	KLK6	NM_001012965:exon5:c.347_351del:p.K116Rfs*60	frameshift deletion	.	.
chr20	ATRN	NM_001207047:exon12:c.1572_1635del:p.T528Gfs*25	frameshift deletion	.	.
chr20	MYT1	NM_004535:exon7:c.G1096A:p.E366K	nonsynonymous SNV	Damaging	Probably damaging
chr21	KRTAP13-1	NM_181599:exon1:c.90_91insAACTGCTGCTCTGGAAACTTCT:p.P31Nfs*16	frameshift insertion	.	.

chrX	NHS	NM_001291868:exon6:c.2730_2731insAGTAAAGA:p.H911Sfs*14	frameshift insertion	.	.
chrX	NHS	NM_001291868:exon6:c.2731_2732insCTTGAACTTCCAATT:p.H911delinsP*	stopgain		
chrX	SSX5	NM_021015:exon2:c.9_10insTAGTAGTGGCCCAGGG:p.D4*	frameshift insertion	.	.
chrX	NLGN3	NM_001166660:exon2:c.C359T:p.P120L	nonsynonymous SNV	Damaging	Probably damaging

chr, chromosome; AA, amino acid; SNV, single nucleotide polymorphism

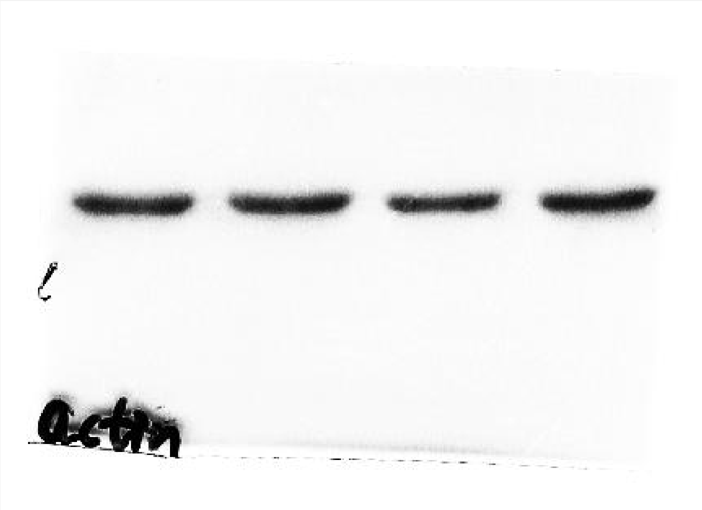

Actin

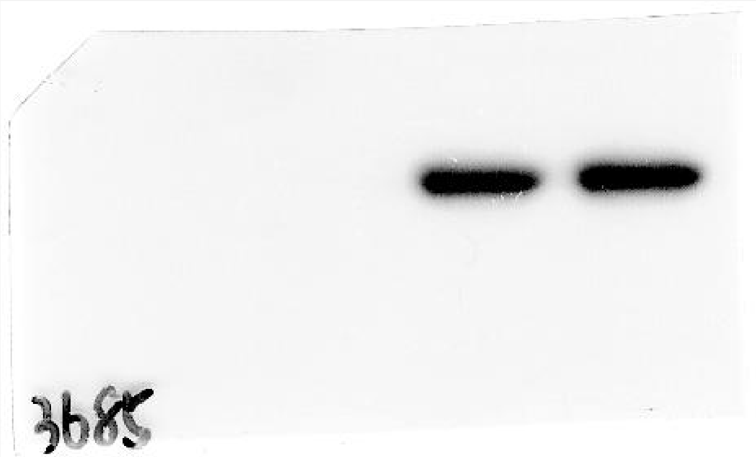

Flag

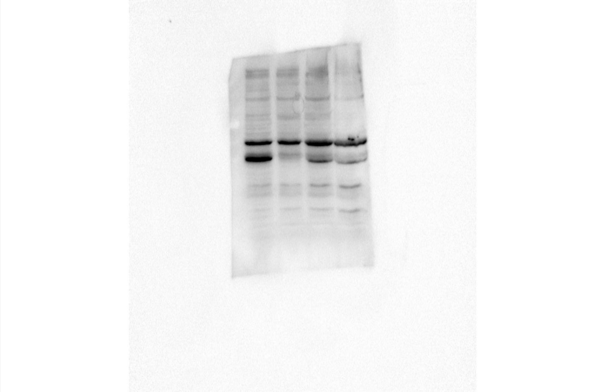

EGFR

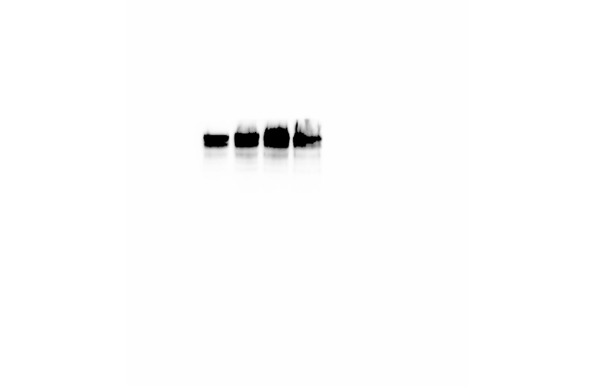

HER2

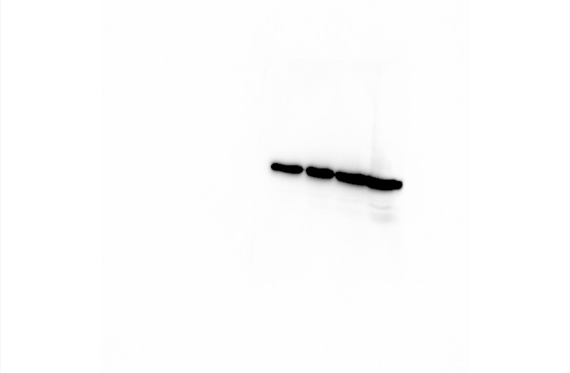

GAPDH

HER2

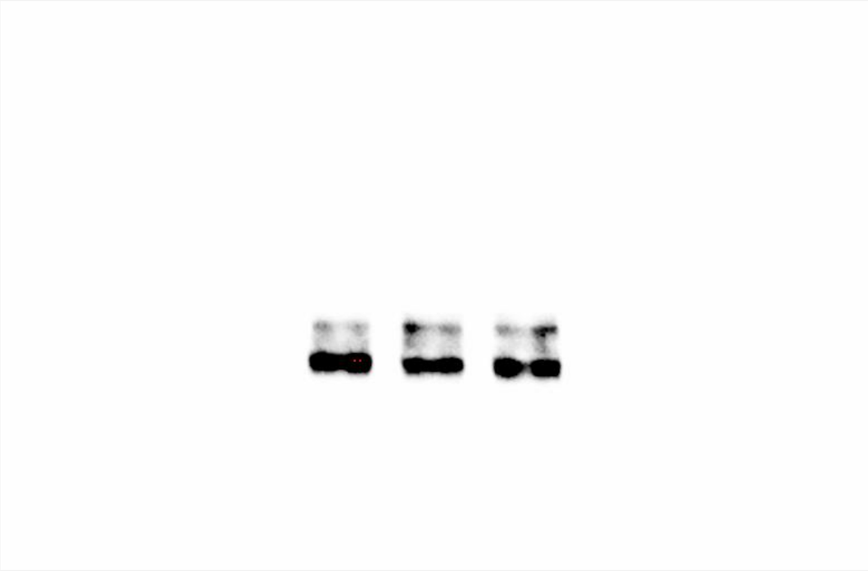

control

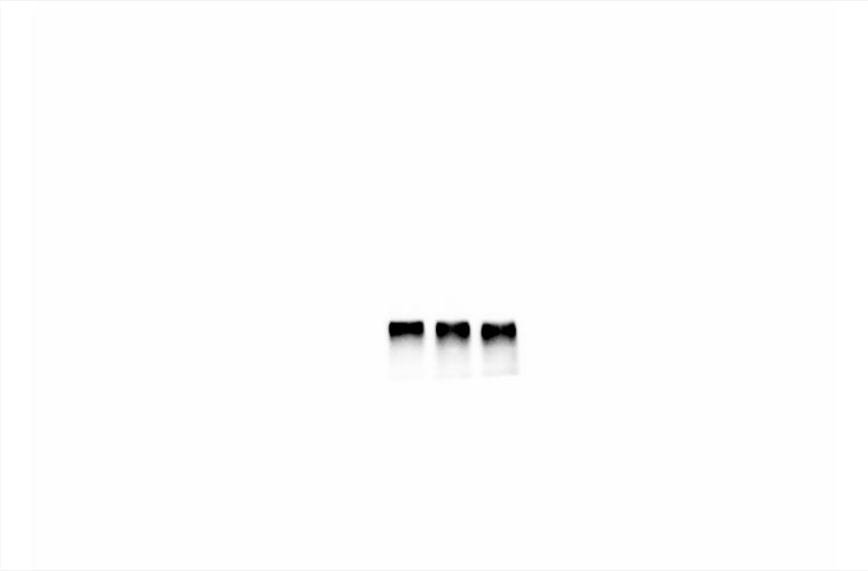

Lapatinib

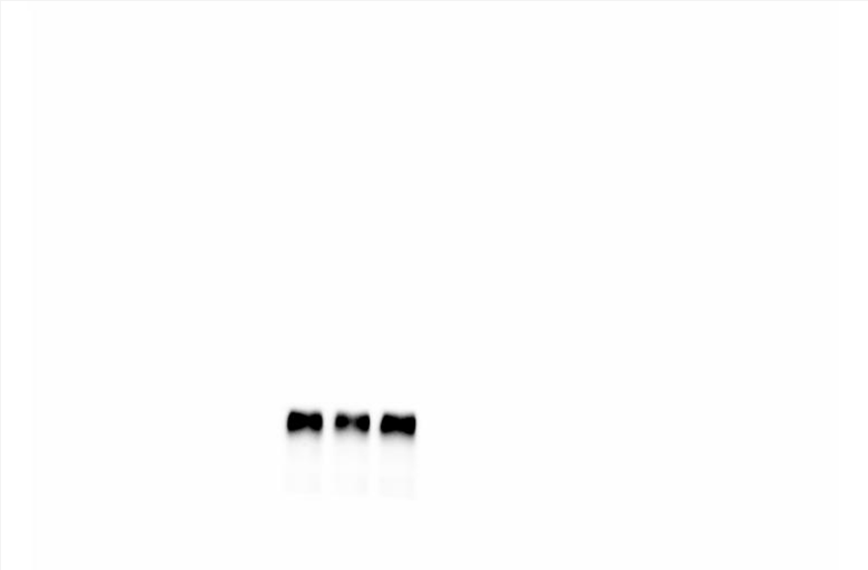

Palbociclib

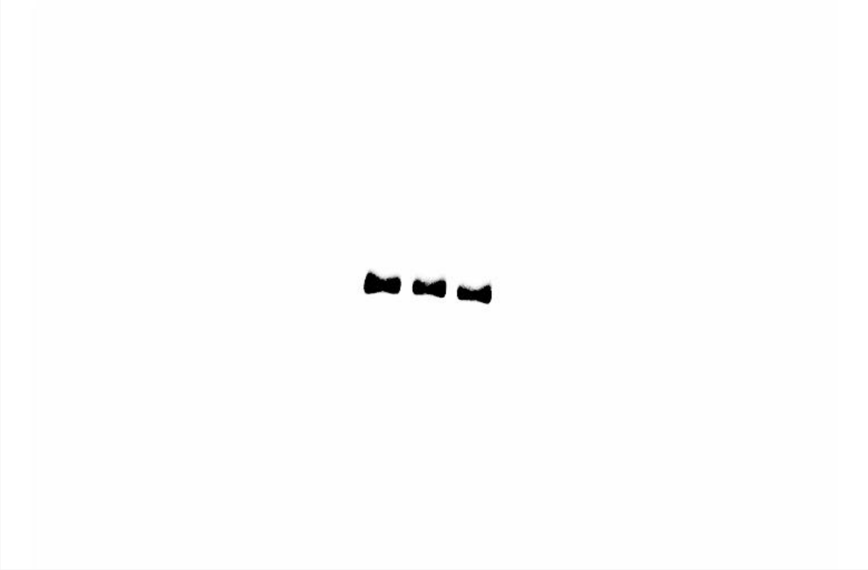

Lap+Pal

p-HER2

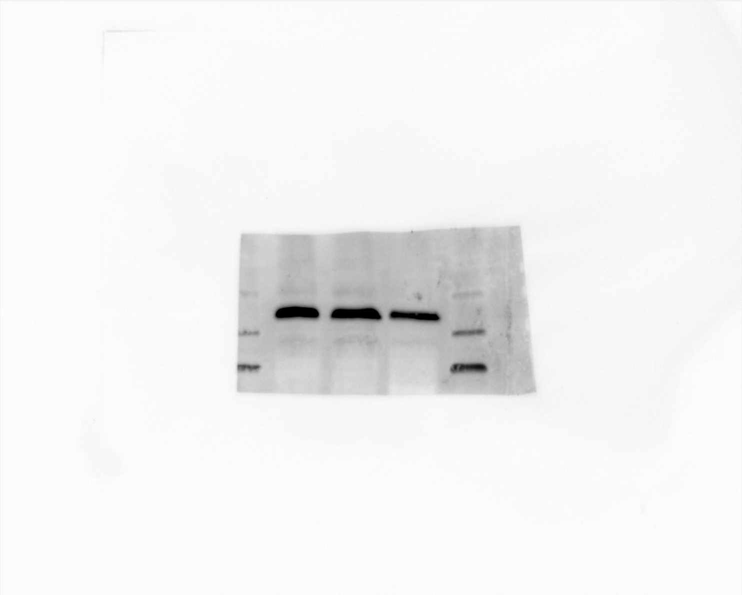

control

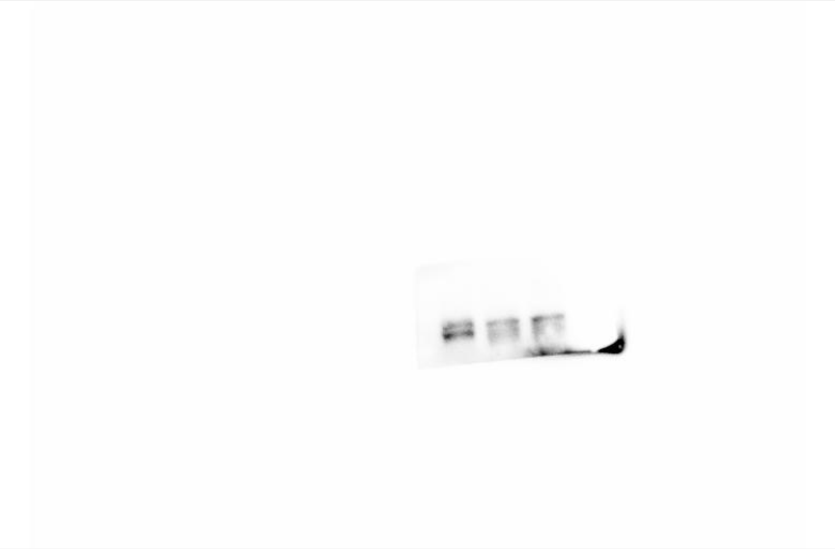

Lapatinib

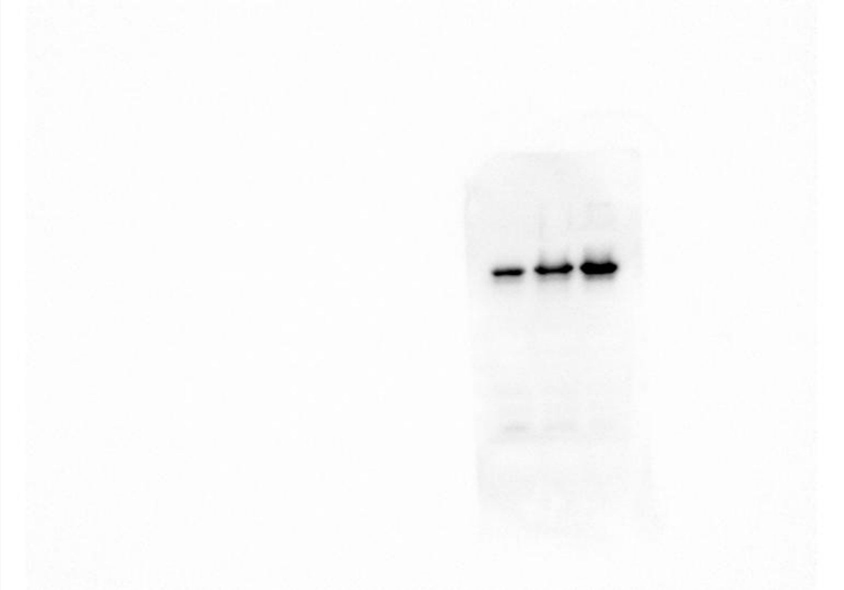

Palbociclib

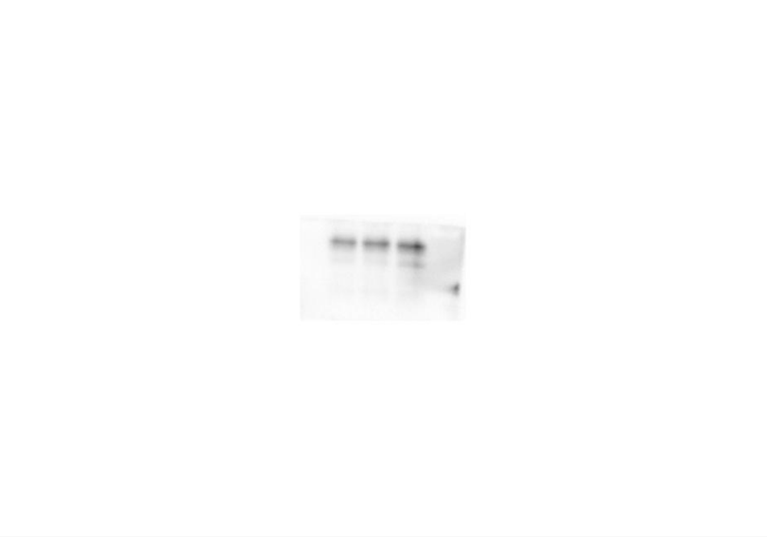

Lap+Pal

EGFR

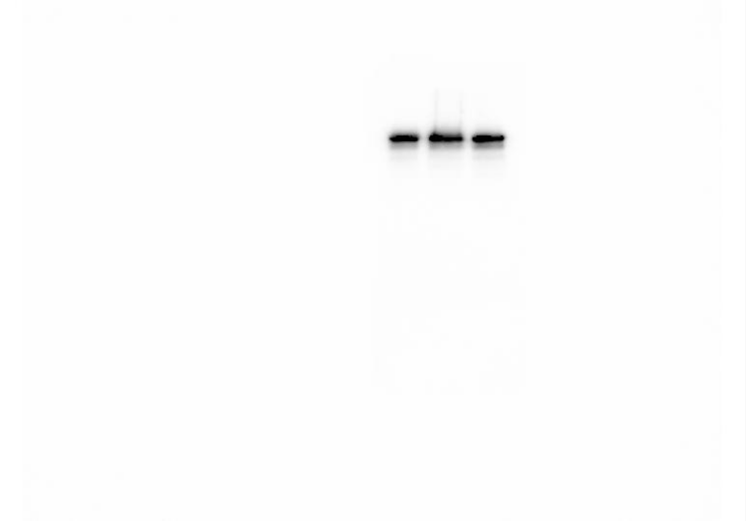

control

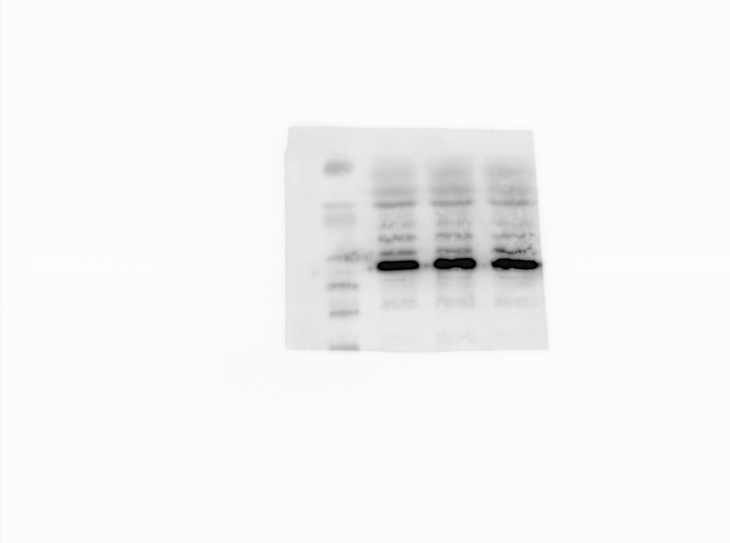

Lapatinib

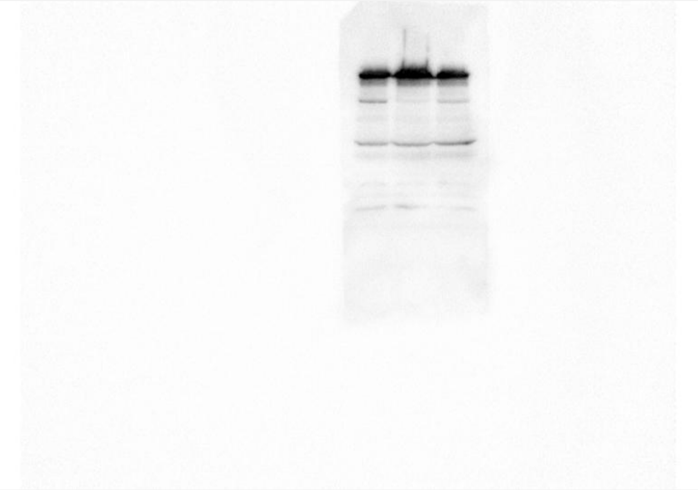

Palbociclib

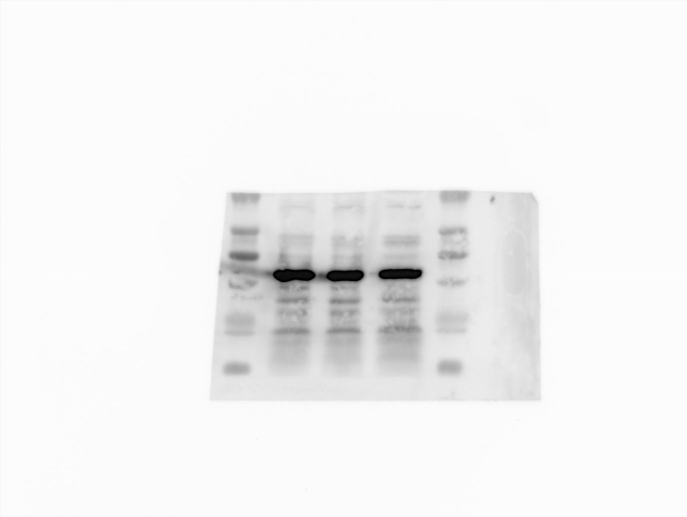

Lap+Pal

p-EGFR

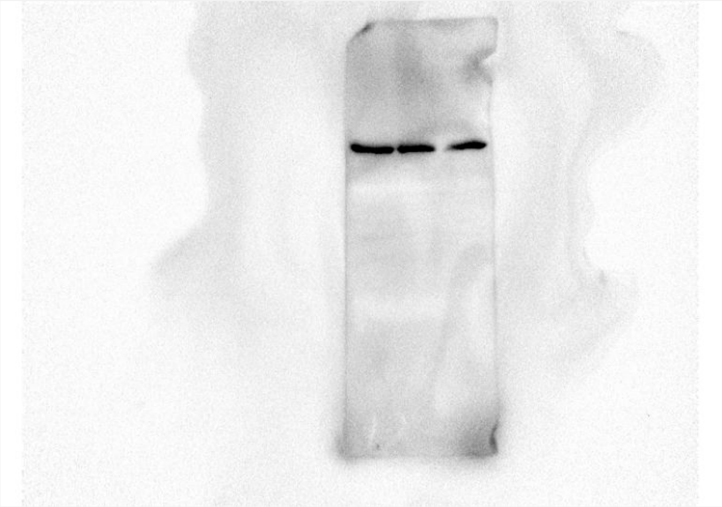

control

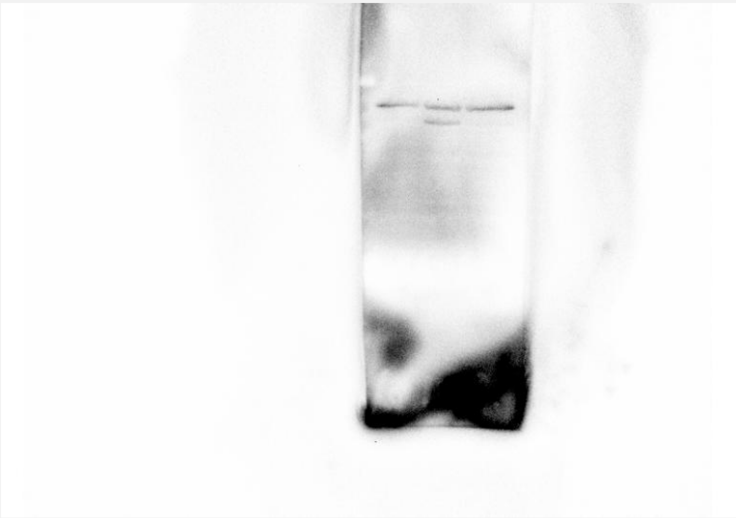

Lapatinib

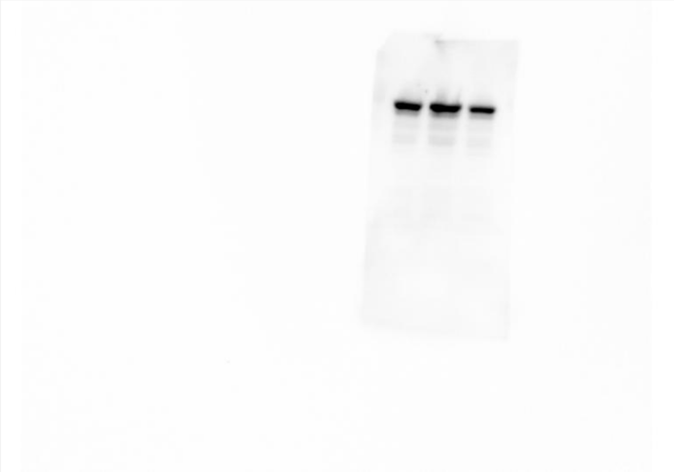

Palbociclib

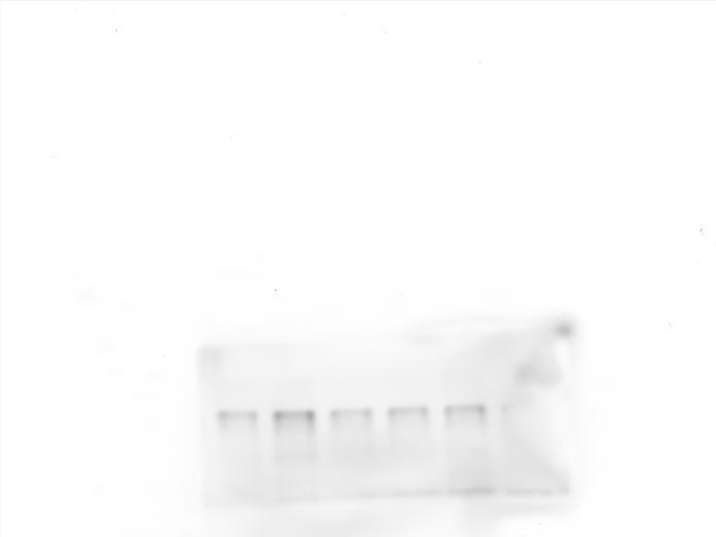

Lap+Pal

TSC2

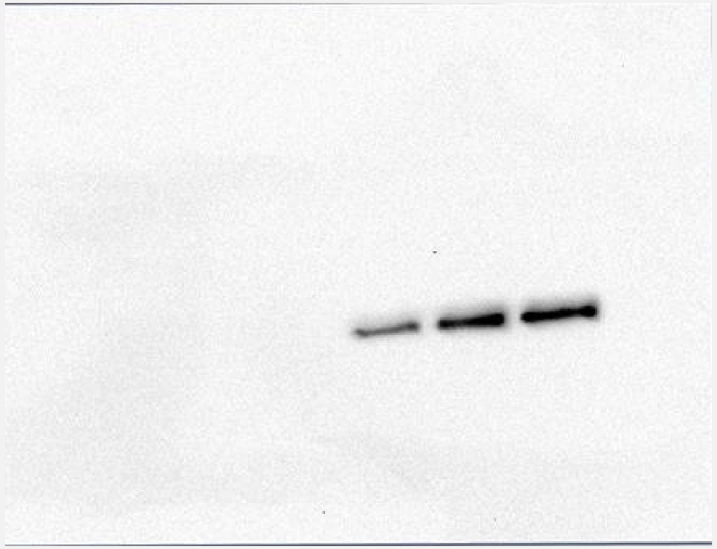

control

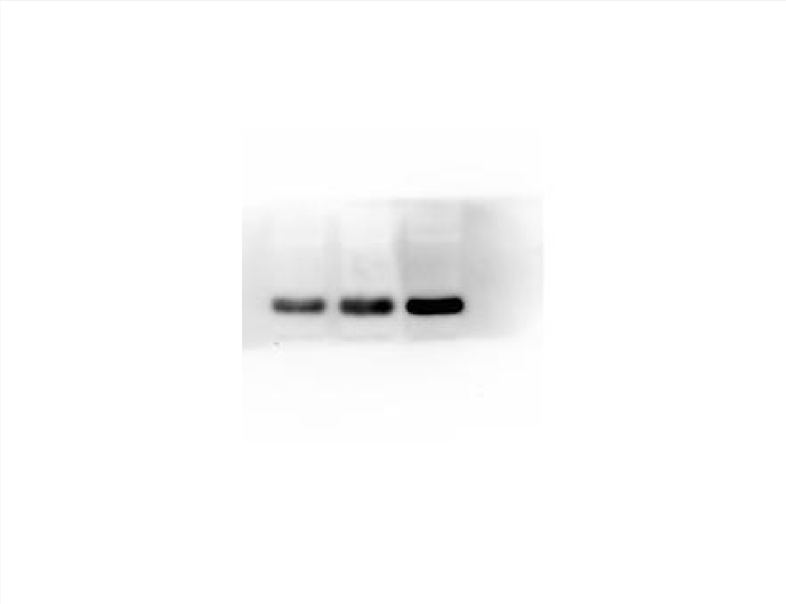

Lapatinib

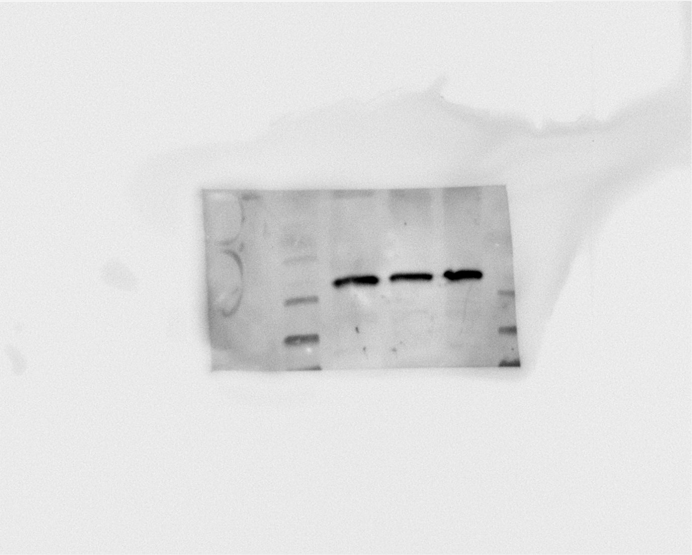

Palbociclib

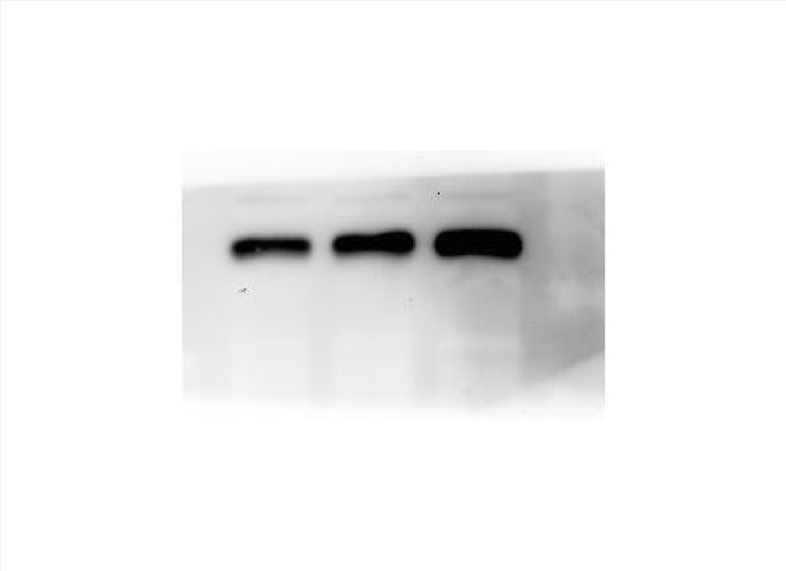

Lap+Pal

p-TSC2

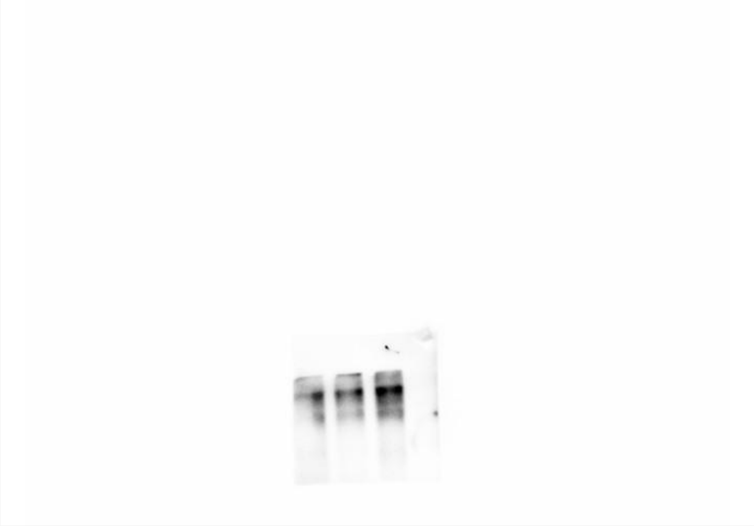

control

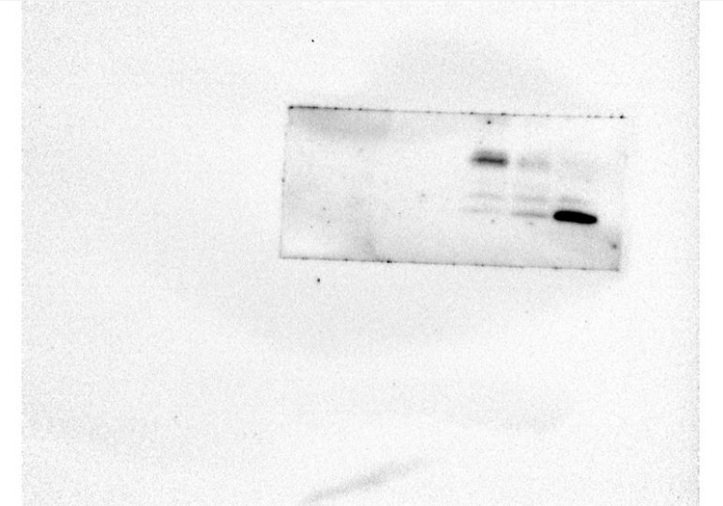

Lapatinib

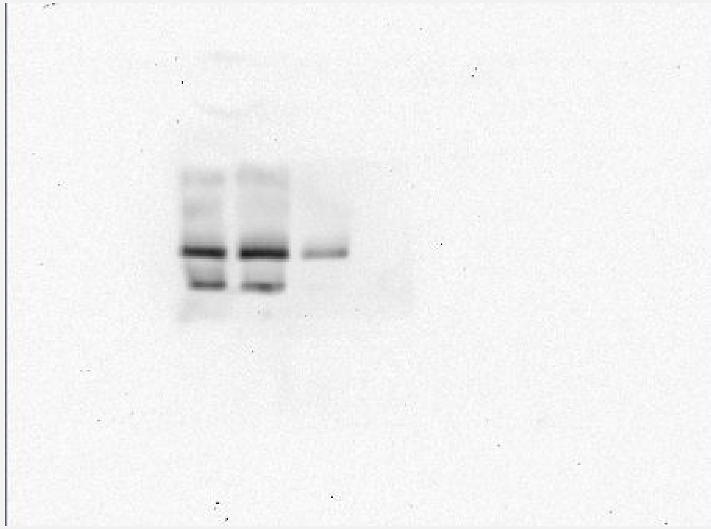

Palbociclib

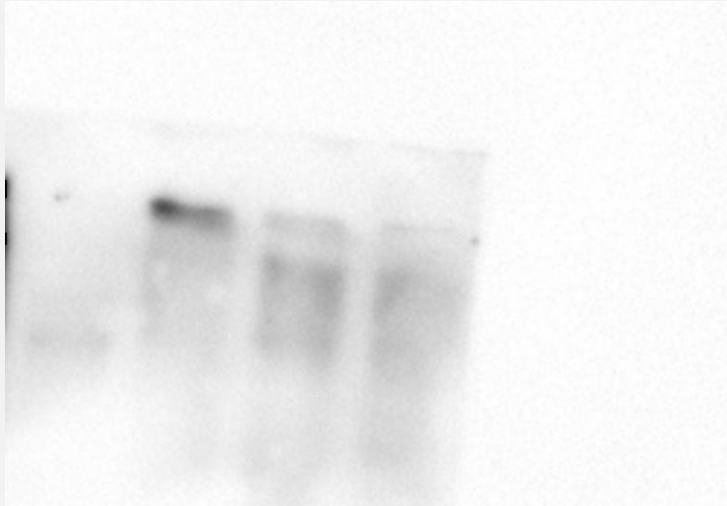

Lap+Pal

mTOR

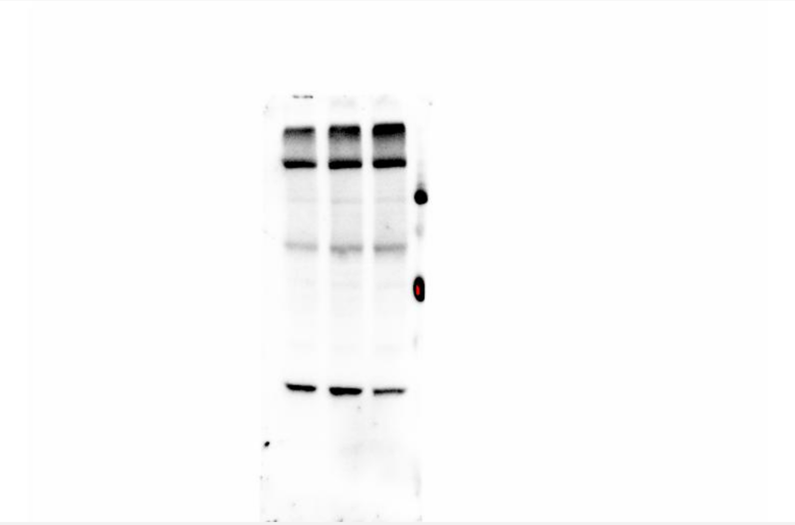

control

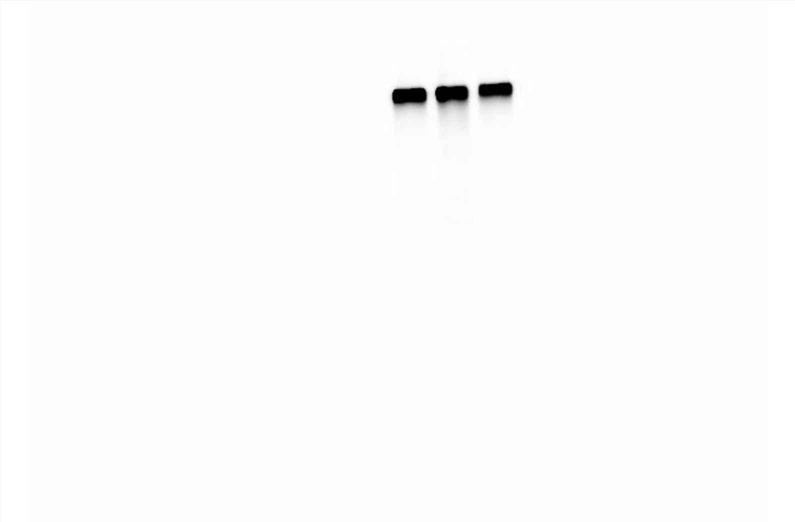

Lapatinib

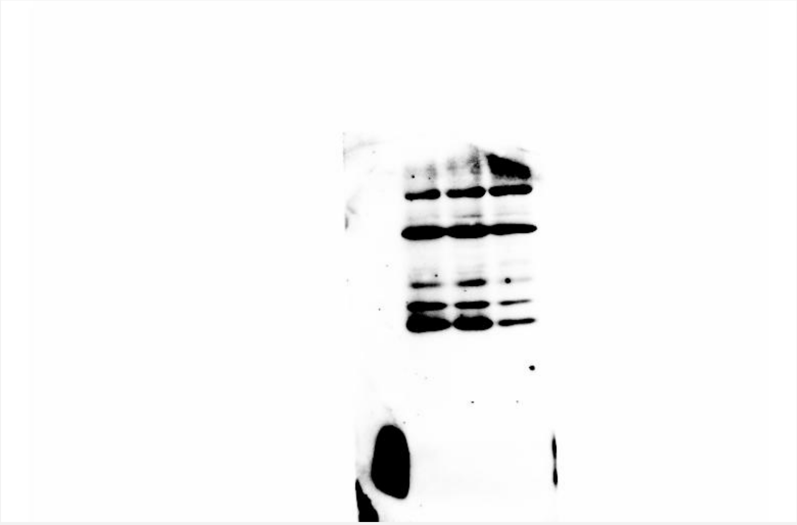

Palbociclib

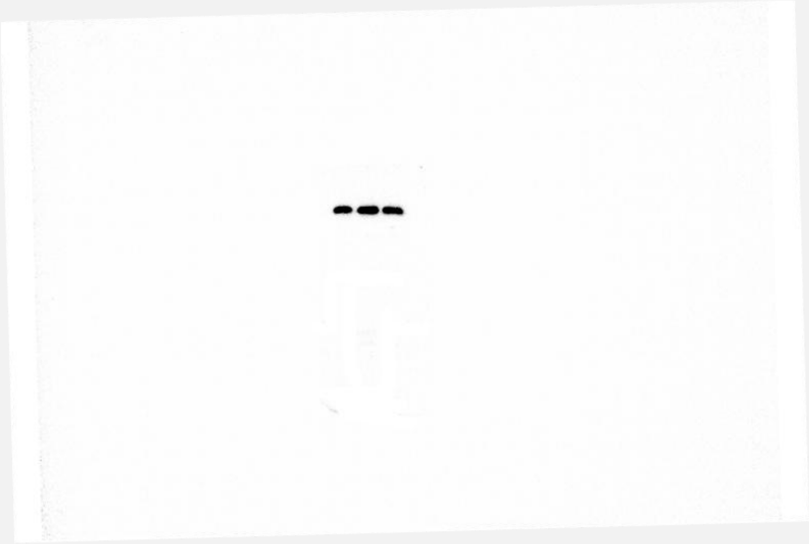

Lap+Pal

p-mTOR

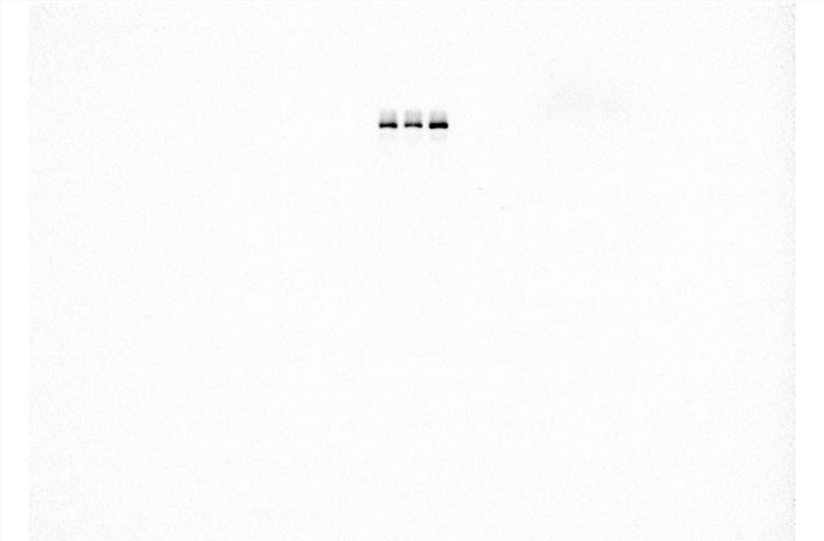

control

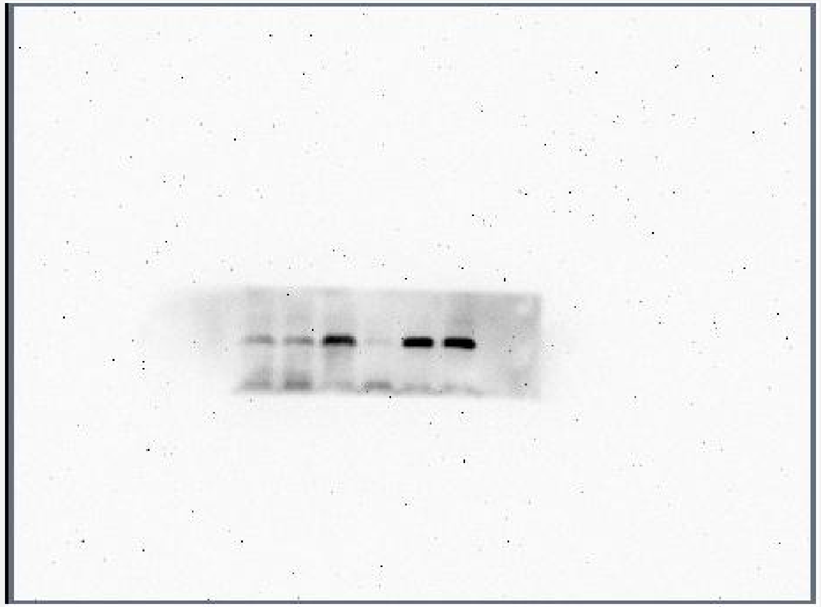

Lapatinib

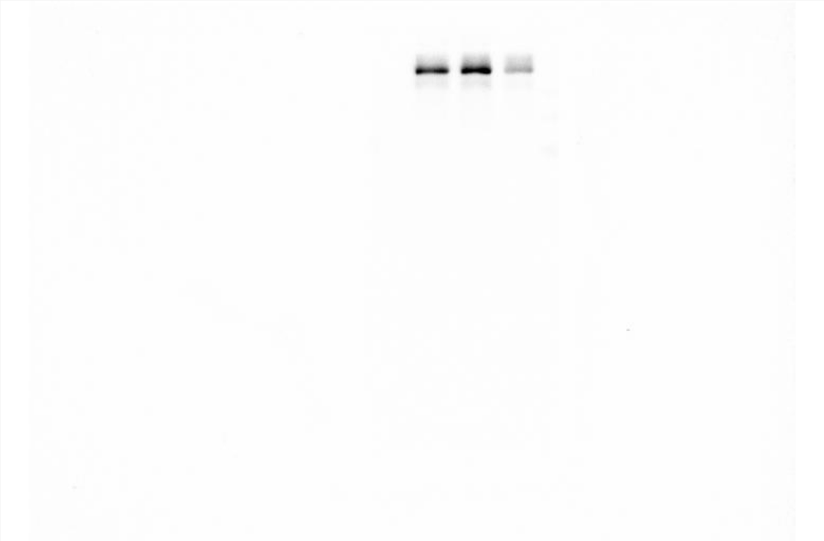

Palbociclib

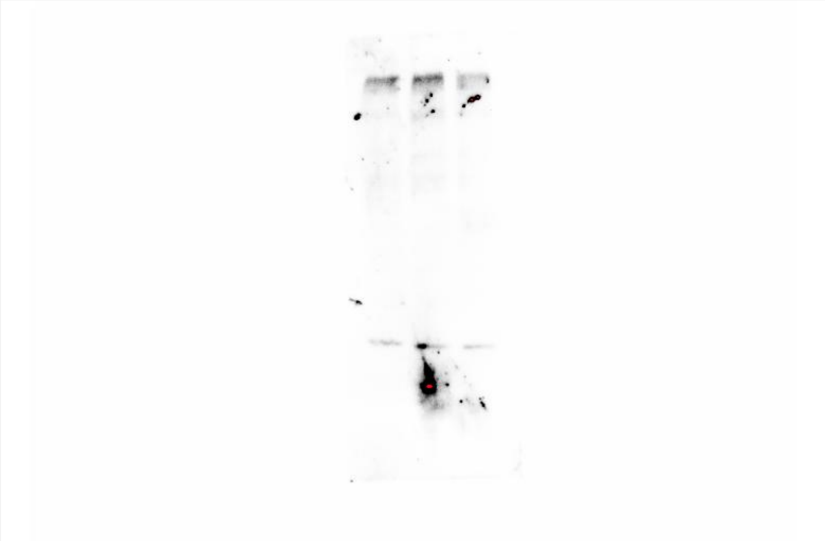

Lap+Pal

P70S6K

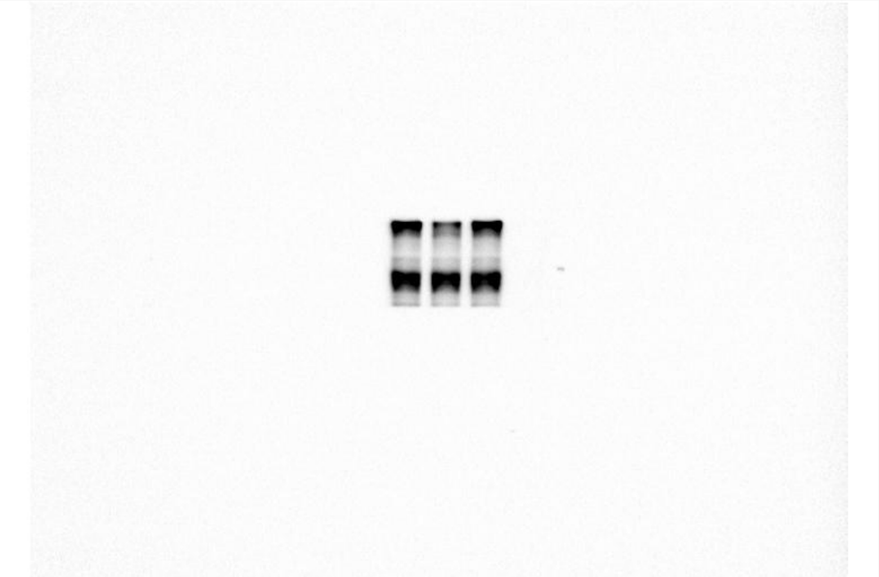

control

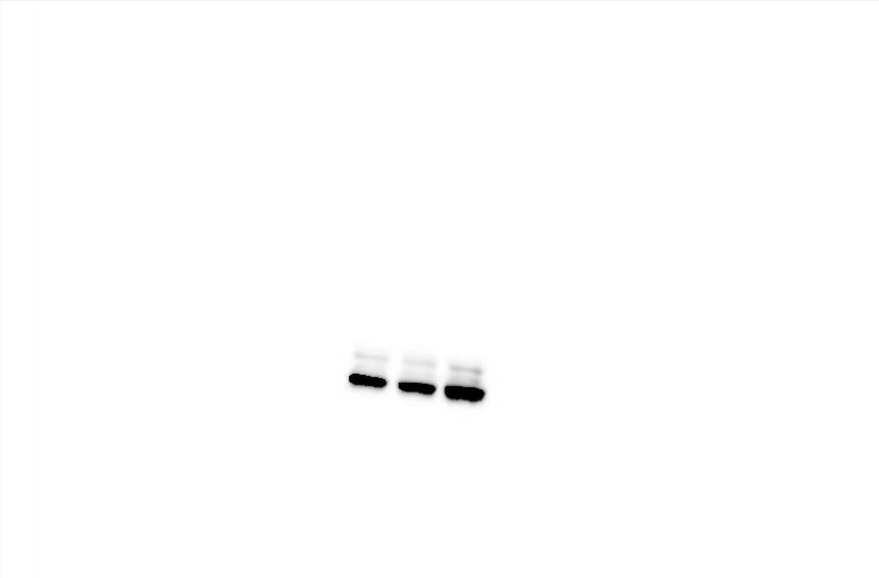

Lapatinib

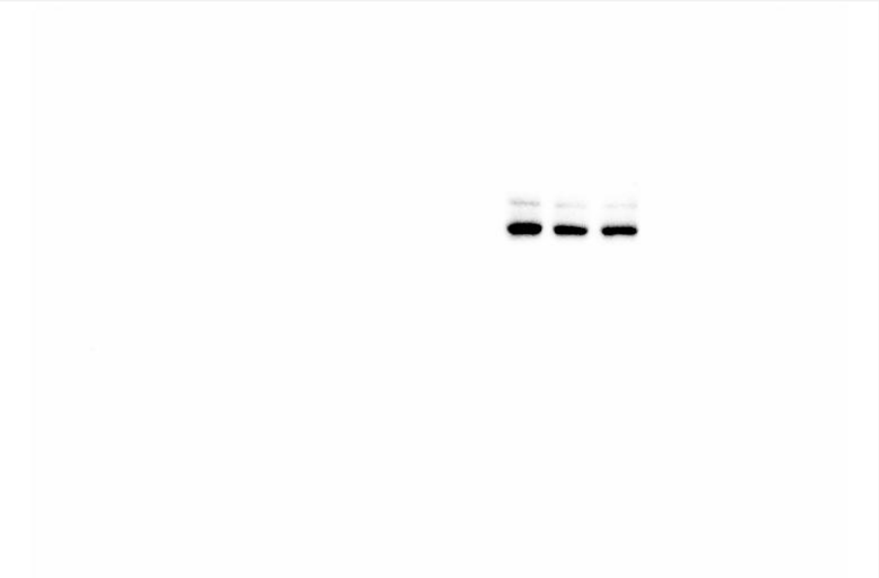

Palbociclib

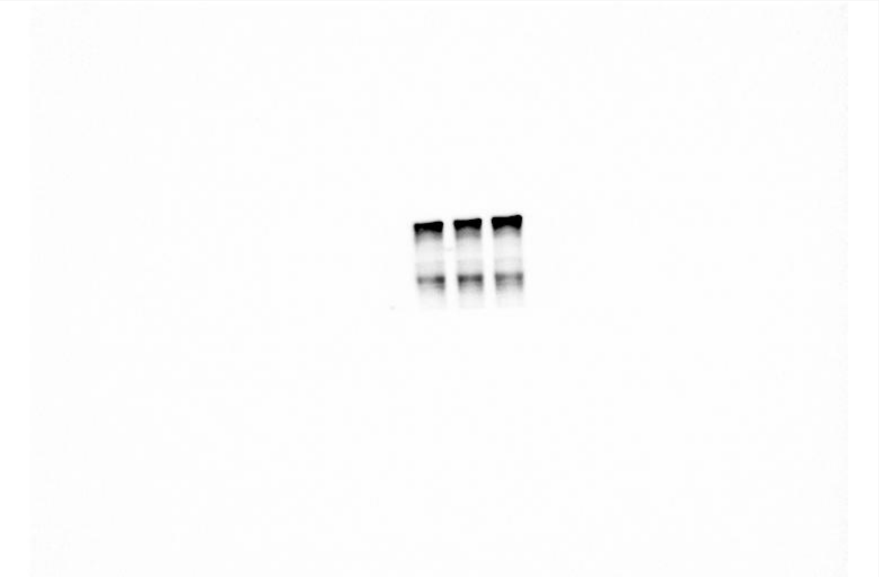

Lap+Pal

p-P70S6K

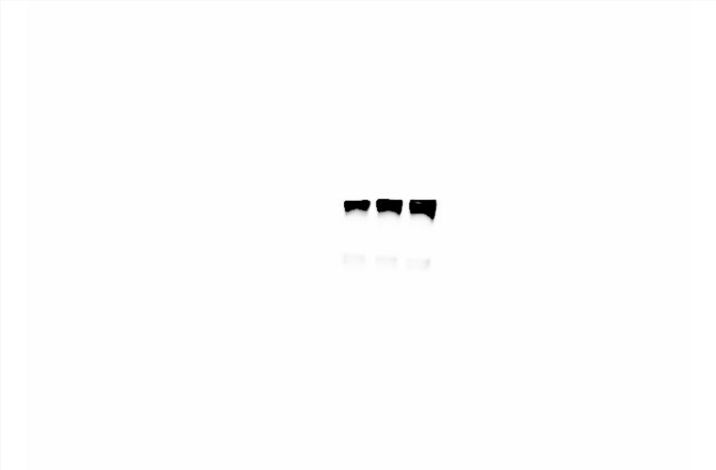

control

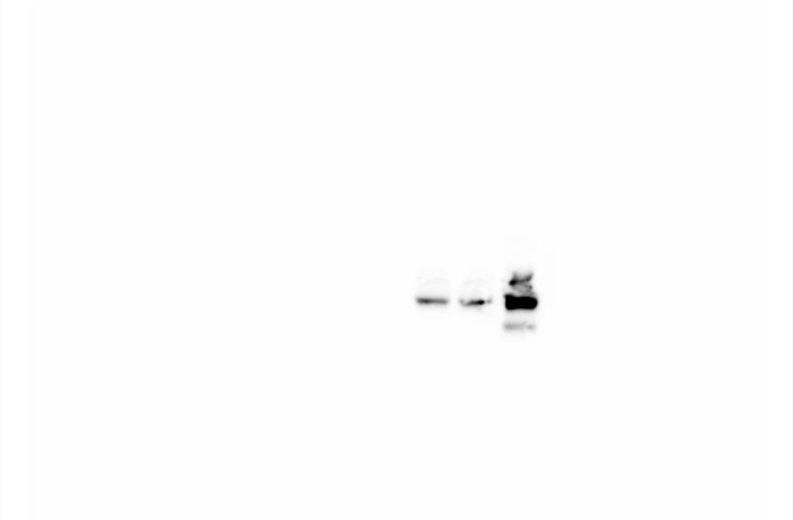

Lapatinib

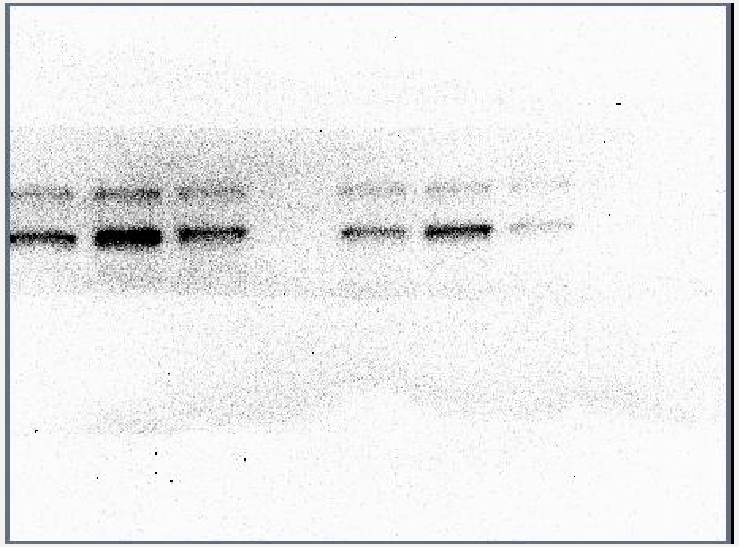

Palbociclib

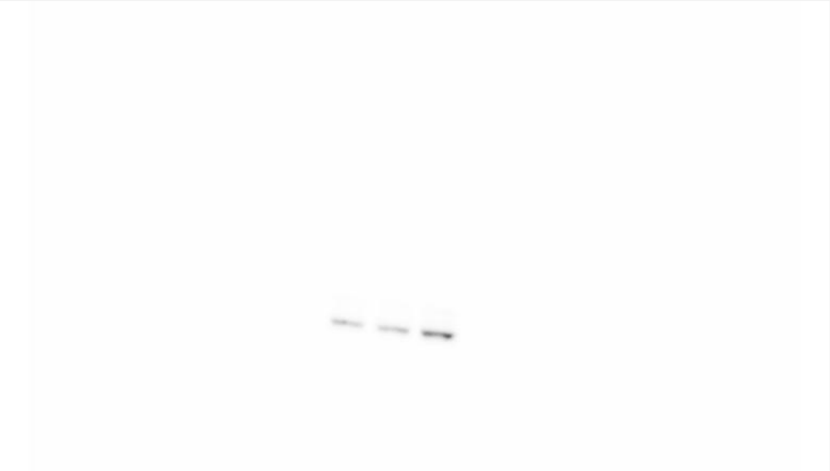

Lap+Pal

CDK4

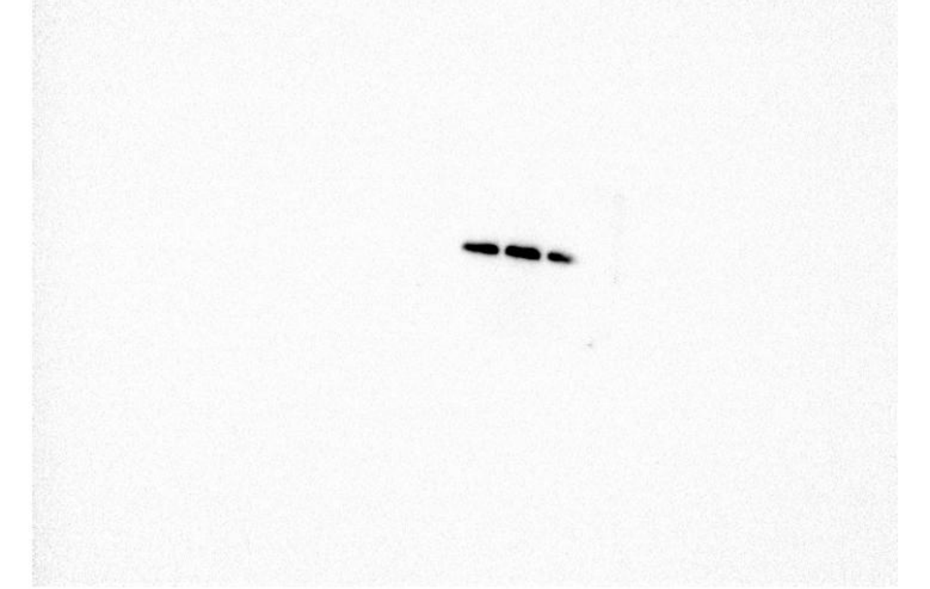

control

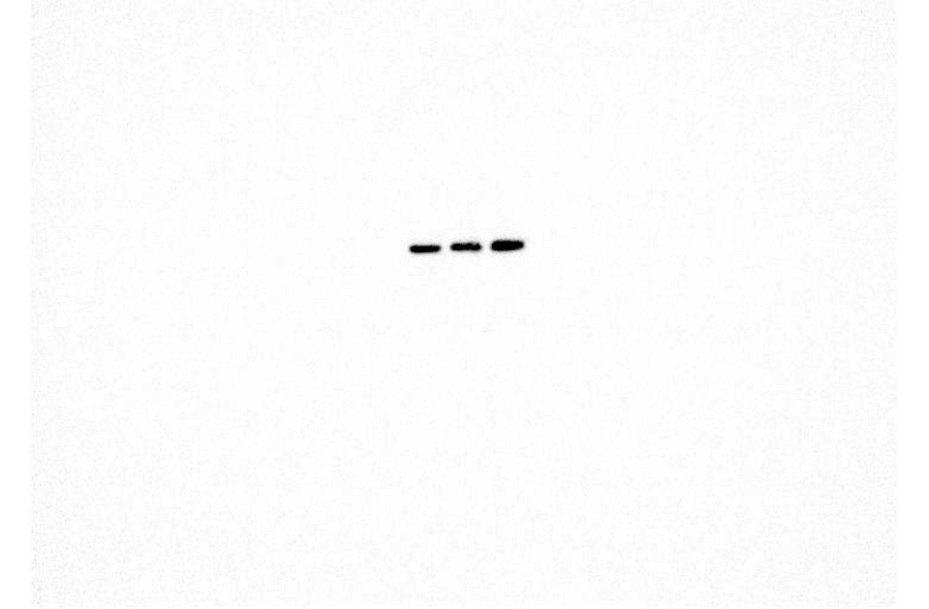

Lapatinib

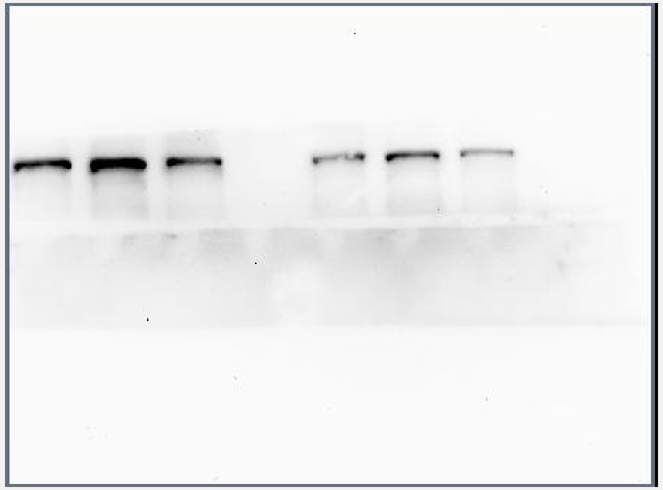

Palbociclib

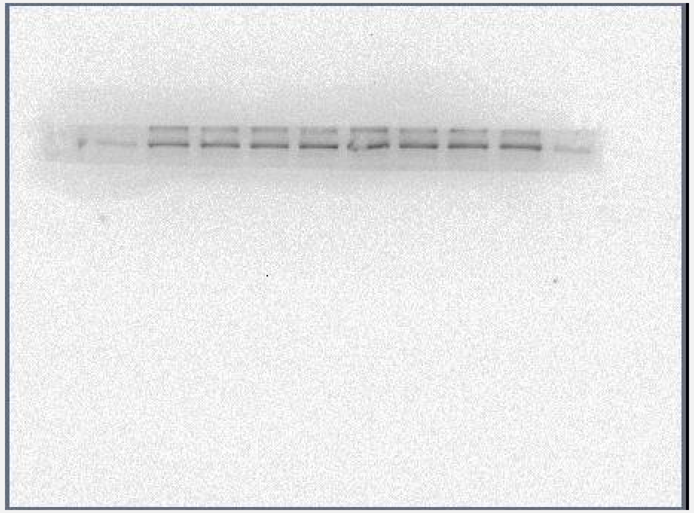

Lap+Pal

RB

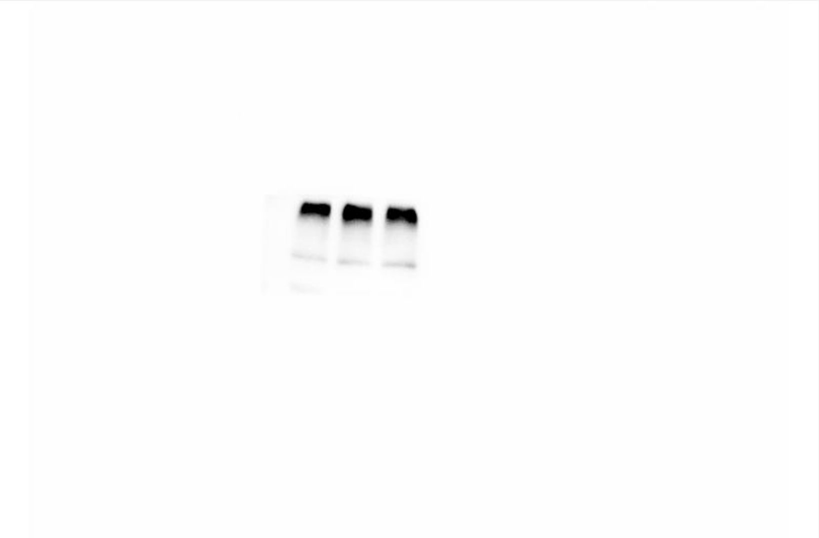

control

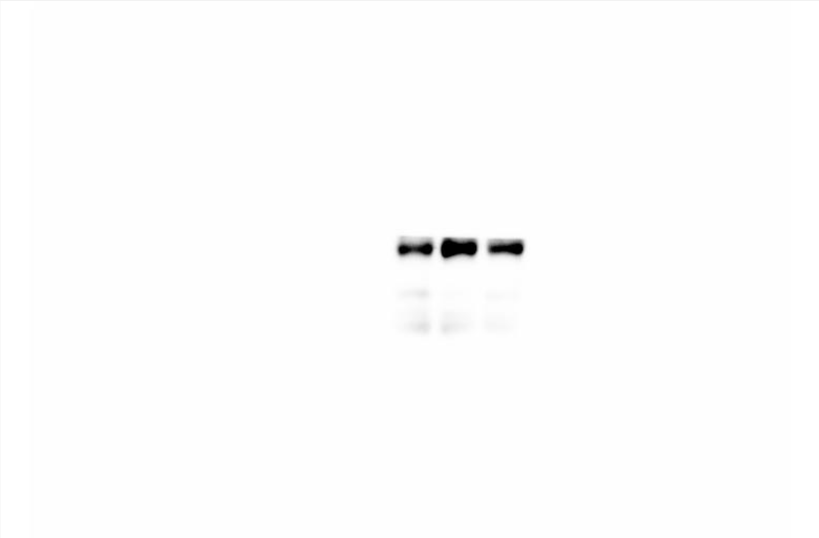

Lapatinib

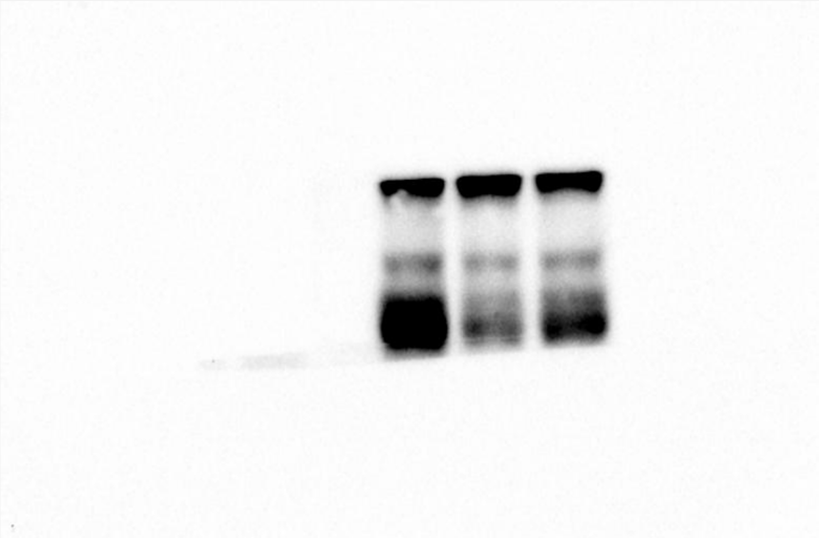

Palbociclib

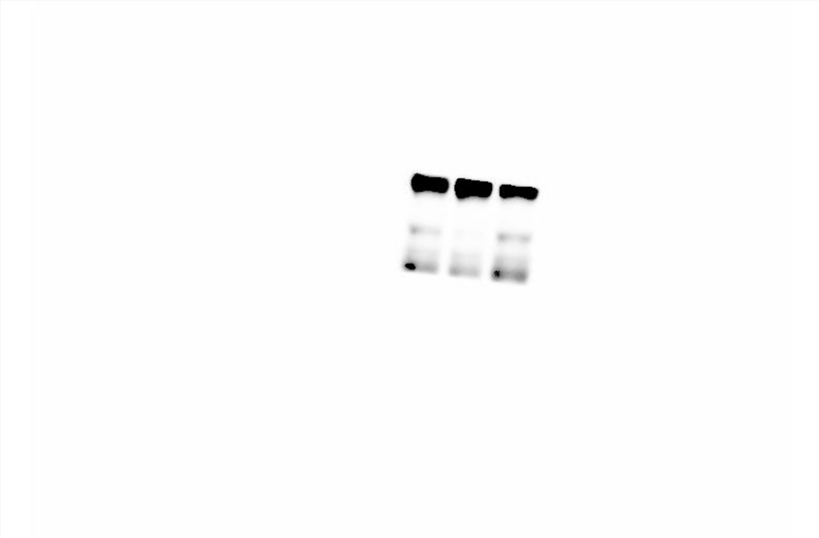

Lap+Pal

p-RB

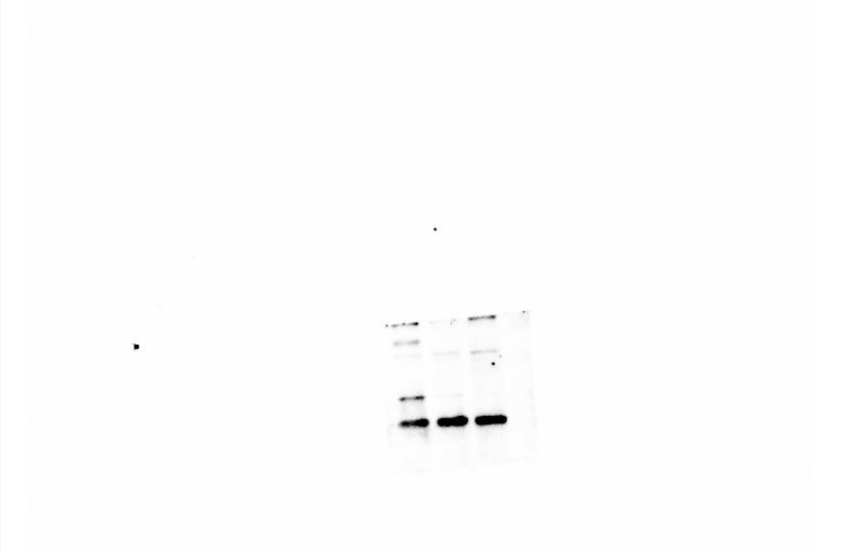

control

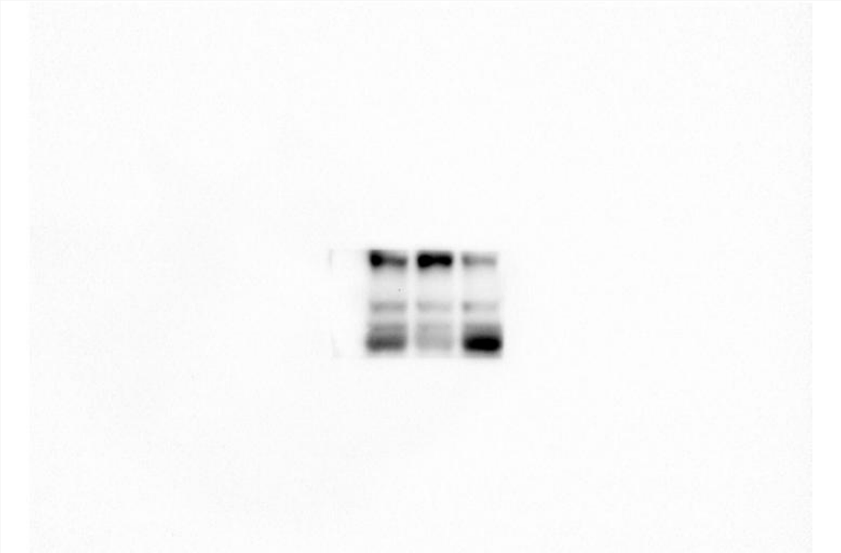

Lapatinib

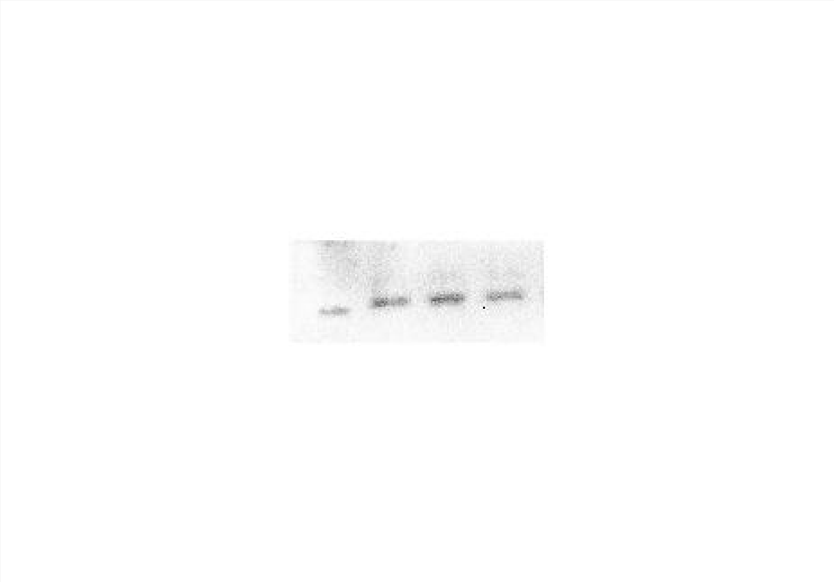

Palbociclib

Lap+Pal

Actin

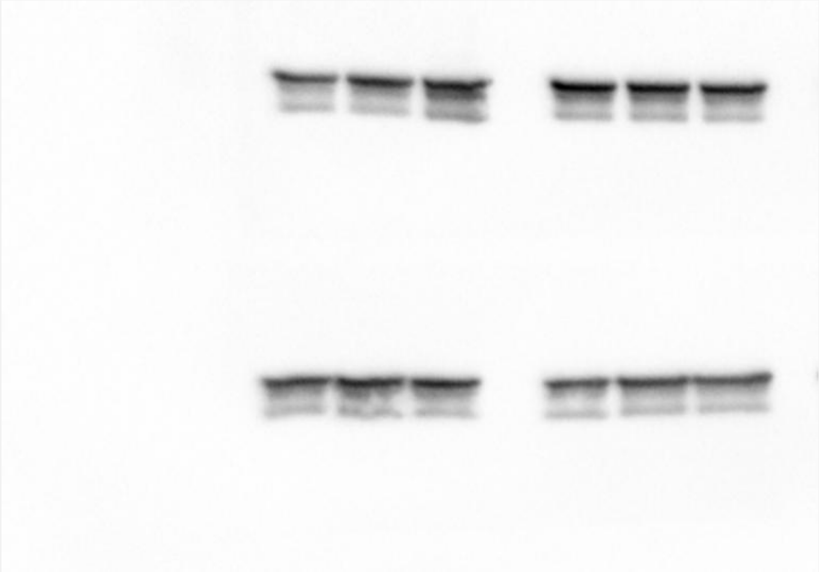

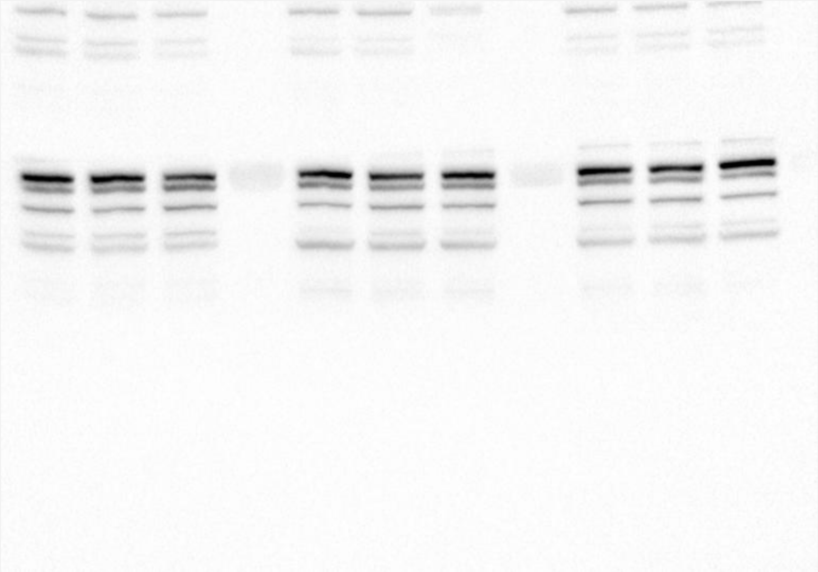

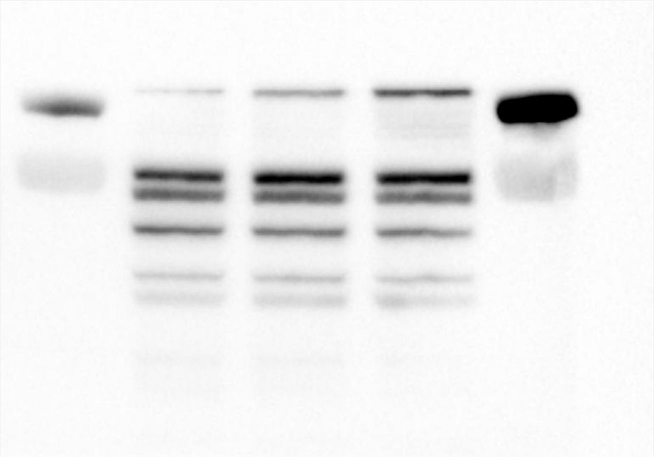

Supplement: Supplementary file 1 — Supplementary Material [file 41523_2023_542_MOESM1_ESM.pdf]
