# Supplementary material for: Resistance to anti-HER2 therapy associated with the TSC2 nonsynonymous variant c.4349 C > G (p.Pro1450Arg) is reversed by CDK4/6 inhibitor in HER2-positive breast cancer
Source: NPJ Breast Cancer. 2023 May 9;9:36. doi: 10.1038/s41523-023-00542-1 (PMC10170158; doi:10.1038/s41523-023-00542-1)
Supplement: Supplementary file 2 — Reporting Summary [file 41523_2023_542_MOESM2_ESM.pdf]

nature portfolio

Corresponding author(s): Wenming Cao, Xiaojia Wang

Last updated by author(s): Feb 22, 2023

Reporting Summary

Nature Portfolio wishes to improve the reproducibility of the work that we publish. This form provides structure for consistency and transparency in reporting. For further information on Nature Portfolio policies, see our Editorial Policies and the Editorial Policy Checklist.

Statistics

For all statistical analyses, confirm that the following items are present in the figure legend, table legend, main text, or Methods section.

n/a	Confirmed	
☐	☒	The exact sample size (*n*) for each experimental group/condition, given as a discrete number and unit of measurement
☐	☒	A statement on whether measurements were taken from distinct samples or whether the same sample was measured repeatedly
☐	☒	The statistical test(s) used AND whether they are one- or two-sided<br>*Only common tests should be described solely by name; describe more complex techniques in the Methods section.*
☐	☒	A description of all covariates tested
☐	☒	A description of any assumptions or corrections, such as tests of normality and adjustment for multiple comparisons
☐	☒	A full description of the statistical parameters including central tendency (e.g. means) or other basic estimates (e.g. regression coefficient) AND variation (e.g. standard deviation) or associated estimates of uncertainty (e.g. confidence intervals)
☐	☒	For null hypothesis testing, the test statistic (e.g. *F*, *t*, *r*) with confidence intervals, effect sizes, degrees of freedom and *P* value noted<br>*Give P values as exact values whenever suitable.*
☒	☐	For Bayesian analysis, information on the choice of priors and Markov chain Monte Carlo settings
☒	☐	For hierarchical and complex designs, identification of the appropriate level for tests and full reporting of outcomes
☒	☐	Estimates of effect sizes (e.g. Cohen's *d*, Pearson's *r*), indicating how they were calculated

Our web collection on statistics for biologists contains articles on many of the points above.

Software and code

Policy information about availability of computer code

Data collection	There is no data collection for this article
Data analysis	There is no specific code in this article. Common statistics methods are mentioned in this article

For manuscripts utilizing custom algorithms or software that are central to the research but not yet described in published literature, software must be made available to editors and reviewers. We strongly encourage code deposition in a community repository (e.g. GitHub). See the Nature Portfolio guidelines for submitting code & software for further information.

Data

Policy information about availability of data

All manuscripts must include a data availability statement. This statement should provide the following information, where applicable:

- Accession codes, unique identifiers, or web links for publicly available datasets
- A description of any restrictions on data availability
- For clinical datasets or third party data, please ensure that the statement adheres to our policy

The datasets generated during and/or analysed during the current study are available from the corresponding author on reasonable request.

March 2021

Human research participants

Policy information about studies involving human research participants and Sex and Gender in Research.

Reporting on sex and gender	The data disaggregated by sex and gender have been provided in the source data, and the consent has been obtained to share the personal level data where these data are used.
Population characteristics	A 33-year-old female patient was diagnosed with stage ?A (cT3N1M0) breast cancer in August 2016. Immunohistochemistry demonstrated the following results: HER2 (3+), ER (+, 20%), PR (−), and Ki-67 (+, 70%). From August 22, 2016 to January 10, 2017, the patient underwent neoadjuvant treatment as follows: AC*4 regimen (doxorubicin + cyclophosphamide) followed by PH*4 regimen (paclitaxel + trastuzumab).
Recruitment	The patient was treated for breast cancer in our hospital, and gene testing was carried out. The patient has gene mutation and the disease cannot be controlled by targeted drugs
Ethics oversight	Medical Ethics Committee of Zhejiang Cancer Hospital

Note that full information on the approval of the study protocol must also be provided in the manuscript.

Field-specific reporting

Please select the one below that is the best fit for your research. If you are not sure, read the appropriate sections before making your selection.

☒ Life sciences ☐ Behavioural & social sciences ☐ Ecological, evolutionary & environmental sciences

For a reference copy of the document with all sections, see nature.com/documents/nr-reporting-summary-flat.pdf

Life sciences study design

All studies must disclose on these points even when the disclosure is negative.

Sample size	In order to detect the drug resistance of TSC2 mutation(TSC2-MT group), NC group and WT group are designed as negative control, so the experiment content is three groups of samples
Data exclusions	No data were excfuded from the analyses
Replication	Confirm that all replication attempts were successful.
Randomization	We randomly selected tumor bearing nude mice for grouping experiment
Blinding	Our experiment is applicable to the experiment that has objective observation indicators and is difficult to achieve blind method. This experiment is not a randomized controlled experiment and does not require a blind method.

Reporting for specific materials, systems and methods

We require information from authors about some types of materials, experimental systems and methods used in many studies. Here, indicate whether each material, system or method listed is relevant to your study. If you are not sure if a list item applies to your research, read the appropriate section before selecting a response.

Materials & experimental systems

n/a	Involved in the study	
☐	☒	Antibodies
☐	☒	Eukaryotic cell lines
☒	☐	Palaeontology and archaeology
☐	☒	Animals and other organisms
☒	☐	Clinical data
☒	☐	Dual use research of concern

Methods

n/a	Involved in the study	
☒	☐	ChIP-seq
☐	☒	Flow cytometry
☒	☐	MRI-based neuroimaging

Antibodies

Antibodies used	rabbit anti-TSC2, obtained from Proteintech, Wuhan, China ,Catalog Number:24601-1-AP, No clone number

Antibodies used

rabbit anti-mTOR, obtained from Proteintech, Wuhan, China, Catalog Number:28657-1-AP,No clone number
rabbit anti-p70S6K, obtained from Proteintech, Wuhan, China, Catalog Number:14485-1-AP, No clone number
mouse anti-actin , obtained from Proteintech, Wuhan, China, Catalog Number:66009-1-Ig ,No clone number
mouse anti-GAPDH obtained from Proteintech, Wuhan, China, Catalog Number: 60004-1-lg. No clone number
Rabbit anti-p-mTOR (Ser2448), obtained from Cell Signaling Technology, Inc, Catalog Number: 5536 , Lot Number: 12
rabbit anti-p-P70S6K (Thr389)(108D2), obtained from Cell Signaling Technology, Inc, Catalog Number: 9234
rabbit anti-CDK4, obtained from Cell Signaling Technology, Inc, Catalog Number: 12790, Lot Number: 5
mouse anti-Rb, obtained from Cell Signaling Technology, Inc. Catalog Number: 9309, Lot Number: 6
rabbit anti-p-Rb (Ser807/811)(D20B12) (obtained from Cell Signaling Technology, Inc.), Catalog Number: 8516 Lot Number: 7
Rabbit anti-p-TSC2 (Thr1462), obtained from Affinity Biosciences, Jiangsu, China, Catalog Number: AF3334
rabbit anti-HER2, obtained from Affinity Biosciences, Jiangsu, China, Catalog Number: AF7681
rabbit anti-p-HER2/ERBB2 (Tyr1248), obtained from Affinity Biosciences, Jiangsu, Catalog Number: AF3069
rabbit anti-EGFR, obtained from Affinity Biosciences, Jiangsu, China, Catalog Number: AF6043
rabbit anti-p-HER1/EGFR (Tyr1173) (obtained from Affinity Biosciences, Jiangsu, China). Catalog Number: AF3048
Mouse anti-EGF Receptor , obtained from Abcam, Inc. Catalog Number:(ab218383)
Rabbit anti-ERBB2/HER2(29D8) obtained from Cell Signaling Technology, Inc. Catalog Number:2165S
anti-rabbit IgG (ab175651, Alexa Fluro 405) and anti-mouse (ab150108, Alexa Fluor 594) of fluorescent staining secondary antibody (Abcam, Cambridge, CB2 0AX, UK)

Validation

rabbit anti-TSC2, obtained from Proteintech, Wuhan, China , Tested Applications:IF, IHC, IP, WB, ELISA, Species Specificity: human, mouse , rat. Web: https://www.ptgcn.com/products/TSC2-Antibody-24601-1-AP.htm Cite: PMID: 32572896.

rabbit anti-mTOR, obtained from Proteintech, Wuhan, China, Tested Applications:IHC, WB, ELISA, Species Specificity: human, Web:https://www.ptgcn.com/products/MTOR-Antibody-28273-1-AP.htm Cite: PMID: 36067312.

rabbit anti-P70S6K, obtained from Proteintech, Wuhan, China, Tested Applications:FC, IF, IHC, IP, WB, ELISA, Species Specificity: human, mouse, rat. Web: https://www.ptgcn.com/products/p70(S6K)-Antibody-14485-1-AP.htm. Cite: PMID: 33177714.

mouse anti-Beta Actin , obtained from Proteintech, Wuhan, China, Tested Applications:FC, IF, IHC, IP, WB, ELISA. Web: https://www.ptgcn.com/products/Pan-Actin-Antibody-66009-1-Ig.htm. Cite: PMID: 32581380.

mouse anti-GAPDH, obtained from Proteintech, Wuhan, China, Tested Applications: FC, IF, IHC, IP, WB, ELISA, Specificity: human, mouse, rat. Web: https://www.ptgcn.com/products/GAPDH-Antibody-60004-1-Ig.htm. Cite: PMID: 31666698

Rabbit anti-p-mTOR (Ser2448), obtained from Cell Signaling Technology, Inc, Tested Applications: IF, IHC, IP, WB, Specificity: human, mouse, rat. Web: https://www.cellsignal.com/products/primary-antibodies/phospho-mtor-ser2448-d9c2-xp-rabbit-mab/5536 Cite: PMID: 35841007.

rabbit anti-p-P70S6K (Thr389)(108D2), obtained from Cell Signaling Technology, Inc, Tested Applications: WB, Specificity: human, mouse, rat. Web: https://www.cellsignal.com/products/primary-antibodies/phospho-p70-s6-kinase-thr389-antibody/9205?site-search-type=Products&N=4294956287&Ntt=p-p70s6k&fromPage=plp. Cite: PMID: 34999917.

rabbit anti-CDK4, obtained from Cell Signaling Technology, Inc, Tested Applications: FC, IF, IHC, WB, Specificity: human, mouse, rat. Web: https://www.cellsignal.com/products/primary-antibodies/cdk4-d9g3e-rabbit-mab/12790. Cite PMID: 35904175.

mouse anti-Rb, obtained from Cell Signaling Technology, Inc. Tested Applications: FC, IF, IHC, IP, WB,IF,ChiP, Specificity: human, mouse, rat ,pig. Web: https://www.cellsignal.com/products/primary-antibodies/rb-d20-rabbit-mab/9313. Cite: PMID: 35604910.

rabbit anti-p-Rb (Ser807/811)(D20B12) (obtained from Cell Signaling Technology, Inc.), Tested Applications: FC, IF, IHC, IP, WB, Specificity: human, mouse, rat. Web: https://www.cellsignal.com/products/primary-antibodies/phospho-rb-ser780-d59b7-rabbit-mab/8180. Cite: PMID: 35395071.

Rabbit anti-p-TSC2 (Thr1462), obtained from Affinity Biosciences, Jiangsu, China, Tested Applications: IF, IHC, WB,ELISA, Specificity: human, mouse, rat. Web: http://www.affbiotech.cn/goods-1495-AF3334-Phospho_Tuberin_TSC2_Thr1462_Antibody.html.

rabbit anti-HER2, obtained from Affinity Biosciences, Jiangsu, China, Tested Applications: WB,IF,ELISA, Specificity: human, mouse, rat. Web: http://www.affbiotech.cn/goods-14327-AF7681-HER2_ErbB2_Antibody.html. Cite: PMID: 30989724

rabbit anti-p-HER2/ERBB2 (Tyr1248), obtained from Affinity Biosciences, Jiangsu, Tested Applications: WB,IF,IHC,ELISA, Specificity: human, mouse, rat. Web: http://www.affbiotech.cn/goods-1240-AF3069-Phospho_HER2_ErbB2_Tyr1248_Antibody.html. Cite: PMID: 30206202.

rabbit anti-EGFR, obtained from Affinity Biosciences, Jiangsu, China, Tested Applications: WB,IF,IHC,ELISA, Specificity: human, mouse, rat. Web: http://www.affbiotech.cn/goods-1717-AF6043-EGFR_Antibody.html. Cite: PMID: 33875643.

rabbit anti-p-HER1/EGFR (Tyr1173) (obtained from Affinity Biosciences, Jiangsu, China). Tested Applications: WB,IF,IHC,ELISA, Specificity: human, mouse, rat. Web: http://www.affbiotech.cn/goods-1221-AF3048-Phospho_EGFR_Tyr1173_Tyr1197_Antibody.html. Cite: PMID: 31173177

Mouse anti-EGF Receptor (ab218383), obtained from Abcam, Inc. Tested Applications: ICC, FC, Specificity: human, Web:https://www.abcam.cn/egfr-antibody-31g7-ab218383.html. Cite: Kim HW et al. Breed- and age-related differences in canine mammary tumors. Can J Vet Res 80:146-55 (2016). PMID: 27127342

Rabbit anti-ERBB2/HER2(29D8) obtained from Cell Signaling Technology, Inc. Tested Applications: WB, IP, IHC, FC, IF, Web: https://www.cellsignal.cn/products/primary-antibodies/her2-erbb2-29d8-rabbit-mab/2165?site-search-type=Products&N=4294956287&Ntt=her2&fromPage=plp. Cite: Wang Q, Bergholz JS, Ding L, et al. STING agonism reprograms tumor-associated macrophages and

overcomes resistance to PARP inhibition in BRCA1-deficient models of breast cancer. Nat Commun. 2022;13(1):3022. Published 2022 May 31. doi:10.1038/s41467-022-30568-1

Eukaryotic cell lines

Policy information about cell lines and Sex and Gender in Research

Cell line source(s)	From ATCC China Cell Bank
Authentication	Cells were STR certified
Mycoplasma contamination	Confirm that all cell lines tested negative for mycoplasma contamination
Commonly misidentified lines (See ICLAC register)	No

Animals and other research organisms

Policy information about studies involving animals; ARRIVE guidelines recommended for reporting animal research, and Sex and Gender in Research

Laboratory animals	Sixty female nude mice (BALB/c) aged 4–6 weeks (Shanghai Slack Laboratory Animal Co., Ltd., SCXK [Shanghai, China] 2017-0005)
Wild animals	The study did not involve wild animals
Reporting on sex	Sixty female nude mice, where this information has been collected in the source data as appropriate
Field-collected samples	All experiments were conducted in accordance with the guidelines of the Administrative Regulations on Laboratory Animal Affairs of Zhejiang University of Traditional Chinese Medicine (Animal Experiment Research Center, Zhejiang University of Traditional Chinese Medicine, SYXK (Zhejiang) 2018-0012) ,The nude mice were fed by specially assigned personnel, grew in a constant temperature environment of 22 degrees, and their weight and tumor volume were measured every 3 days. After 21 days, the mice were euthanized, and tumor specimens were collected.
Ethics oversight	This study approved by the Experimental Animal Ethics Committee.

Note that full information on the approval of the study protocol must also be provided in the manuscript.

Flow Cytometry

Plots

Confirm that:

- [x] The axis labels state the marker and fluorochrome used (e.g. CD4-FITC).
- [x] The axis scales are clearly visible. Include numbers along axes only for bottom left plot of group (a 'group' is an analysis of identical markers).
- [ ] All plots are contour plots with outliers or pseudocolor plots.
- [x] A numerical value for number of cells or percentage (with statistics) is provided.

Methodology

Sample preparation	TSC2-NC, WT and MT cells were suspended in flow buffer (PBS containing 2% fetal bovine serum), and incubated with anti-EGFR (Abcam, Cambridge, CB2 0AX, UK) and anti-HER2 (Cell Signaling Technology, Inc. Danvers, MA 01923, USA) for 2 hours at 4°C in the dark, with a subsequent wash in flow buffer, spin and resuspension. Cells were then fixed, permeabilized and stained with anti-rabbit IgG (ab175651, Alexa Fluro 405) and anti-mouse (ab150108, Alexa Fluor 594) of fluorescent staining secondary antibody (Abcam, Cambridge, CB2 0AX, UK) for 30min at 4°C in the dark at each step. After staining, cells were analyzed using BD CantoII flow cytometer (Becton Dickinson, San Jose, CA, USA).
Instrument	BD CantoII flow cytometer (Becton Dickinson, San Jose, CA, USA).
Software	NovoCyte software and FlowJo analysis software
Cell population abundance	In the cells of TSC2-NC, WT and MT, there are surface markers of EGFR and HER2, which are combined with the surface markers overnight by adding antibodies of EGFR and HER2, and then stained with anti-rabbit IgG (ab175651, Alexa Fluro 488) and anti-mouse (ab150108, Alexa Fluor 594) of fluorescent staining secondary antibody. Distinguish light of different wavelengths received by different channels of flow cytometry. Alexandra Fluro 405 wavelength is 405-421nm, representing PB450 channel. Alexa Fluro 594 wavelength is 594-615nm, representing ECD channel.
Gating strategy	Because EGFR and HER2 are transmembrane proteins, this cell group has been fixed by membrane breaking, so it is

Gating strategy

unnecessary to distinguish between dead and alive cells, Different wavelengths I scatter through different markers.

☒ Tick this box to confirm that a figure exemplifying the gating strategy is provided in the Supplementary Information.
